# Antioxidant, antiaging and mitochondrial protective effects of JadeAging in *Caenorhabditis elegans*

**DOI:** 10.3389/fphar.2025.1644921

**Published:** 2026-01-12

**Authors:** Bowen Lu, Dongli Yin, Jiahao Zhang, Yilei Wang, Xue Wang, Shengcan Zou, Jiacheng Li

**Affiliations:** 1 The First Clinical Medical School, Shandong University of Traditional Chinese Medicine, Jinan, Shandong, China; 2 Qingdao Chenland Pharmaceutical Co., Ltd., Qingdao, Shandong, China; 3 Affiliated Hospital of Shandong University of Traditional Chinese Medicine, Jinan, Shandong, China

**Keywords:** jadeaging, antioxidant, anti-aging, mitochondrial health, *Caenorhabditis elegans*, TOR pathway

## Abstract

**Objective:**

This study aims to evaluate the antioxidant, anti-aging, and mitochondrial protective effects of JadeAging, a Chinese medicine-based longevity formulation, using the model organism *Caenorhabditis elegans*.

**Methods:**

We used *C. elegans* to assess the impact of JadeAging and its individual components (rehmannia, poria, and ginseng) on various health metrics. RNA sequencing (RNA-seq) was performed to identify differentially expressed genes and their effects on biological pathways. Lifespan and healthspan assays were performed to evaluate cell vitality, whereas mitochondrial health was assessed using mitochondrial fragmentation analysis. Antioxidant capacity was determined by measuring protection against reactive oxygen species

**Results:**

Treatment with JadeAging did not significantly extend lifespan but showed improvements in healthspan, with treated animals exhibiting more vigorous movement over their lifespan. JadeAging and its components demonstrated significant antioxidant capacity, protecting *C. elegans* from the deleterious effects of paraquat-induced oxidative stress. Furthermore, JadeAging treatment significantly reduced mitochondrial fragmentation, indicating enhanced mitochondrial health. RNA-Seq data revealed downregulation of daf-2 and rsks-1 and upregulation of cpr-1, suggesting the activation of the Target of Rapamycin pathway and promotion of autophagy.

**Conclusion:**

JadeAging exerts protective effects against oxidative stress and age-related mitochondrial decline, likely by modulating key longevity pathways. These findings support the positive effects of JadeAging and its components on health.

## 1 Introduction

Aging is a multifaceted biological process that is shaped by genetic, epigenetic, environmental, and social factors. It is characterized by a gradual decline in physical functions and a heightened risk of chronic diseases such as neurodegenerative disorders, cardiovascular diseases, and cancer (Sen et al., 2016). These illnesses are among the most common illnesses globally and, contribute significantly to overall morbidity and mortality rates (Kennedy et al., 2014). Oxidative stress is a key mechanism underlying cellular senescence and increased frailty, leading to various age-related, noncommunicable diseases (Martemucci et al., 2022). Mitochondria are crucial for this process because of their roles in the regulationregulating of bioenergetics, oxidative stress, and cell death. Mitochondrial oxidative stress, linked to neurodegenerative disorders, cardiovascular diseases, endocrine dysfunctions, diabetes, and cancer, can damage mitochondrial DNA, proteins, and lipids (Tsang et al., 2022). Elevated levels of reactive oxygen species (ROS) also trigger chronic inflammation, a common feature of several age-related autoimmune diseases. Natural plant extracts have gained popularity in the anti-aging field of their high safety, effectiveness, and minimal side effects (Vaiserman et al., 2019).

JadeAging, a Chinese medicine-based longevity formulation, compriosesd three main active ingredients: Rehmanniae Radix, Poria, and Ginseng Radix. Rehmanniae Radix is a perennial herb belongings to the family Scrophulariaceae. Rehmannia glutinosa (Kim et al., 2017) has significant anti-inflammatory and antioxidant properties, lowers blood sugar levels, improves cognitive function and memory, has anti-depressant effects, and provides protection against hypertension and related kidney damage. In addition, *R. glutinosa* regulates the immune system, enhances the activity of natural killer cells and macrophages, promotes liver cell regeneration, and reduces liver inflammation. *Poria cocos* (Zhang et al., 2021) ^]^, a widely used medicinal fungus, exhibits significant anti-inflammatory properties, particularly by inhibiting the production of nitric oxide (NO) in lipopolysaccharide (LPS)-induced RAW 264.7 cells and reducing the production of inflammation-related cytokines such as TNF-α, IL-1β, and IL-6. The triterpenoid compounds in *Poria cocos* extract, such as poricoic acid B, demonstrate higher anti-inflammatory activity than poricoic acid A, whereas dehydrotrametenolic acid and dehydroeburicoic acid showed no anti-inflammatory activity. These findings suggest that the epidermis of *P. cocos* can be used as a natural anti-inflammatory agent for use in pharmaceuticals and functional foods. Ginseng (Liu et al., 2020), the root of Panax ginseng, has significant anti-inflammatory, antioxidant, antitumor, cardiovascular, nervous system protective, and antidiabetic effects. Its main active components, ginsenosides, inhibit the production of inflammatory factors, protect the heart from ischemia-reperfusion injury, improve cognitive function, suppress the growth and metastasis of tumor cells, and lower blood sugar levels by regulating the expression of glucose transportersg. Overall, ginseng exerts a wide range of pharmacological effects through its various bioactive components, demonstrating its potential to treat a variety of diseases.

*Caenorhabditis elegans* is a well-established model for studying aging because of its ease of cultivation, short lifecycle, evolutionary conservation, and high genetic similarity with humans (Yu et al., 2018). Researchers have frequently used *C. elegans* to screen natural products for anti-aging properties and investigate the mechanisms of aging and stress resistance (Son et al., 2019). Three signaling pathways are crucial for nematode physiological functions: insulin/IGF-1, c-Jun N-terminal kinase (JNK) pathway, and the PMK-1 protein kinase pathways. These highly conserved pathways, which correspond to human and mammalian signaling pathways, play a significant role in regulating longevity and stress responses (Khan et al., 2019). Oxidative stress is a key factor limiting the longevity of both *C. elegans* and humans (Finkel and Holbrook, 2000). Dietary restriction (DR) extends the mammalian lifespans and has shown similar effects in *C. elegans*. Thus, using *C. elegans* as a model for aging research can offer valuable insights into human biology and aid in the evaluation of active compounds for anti-aging and chronic disease treatments (Ye et al., 2020).

In this study, *C. elegans* was used as a model organism to evaluate the effects of the JadeAging formulation and its individual components (rehmannia, poria, and ginseng) on gene expression and various health metrics. RNA sequencing (RNA-seq) was used to identify differentially expressed genes (DEGs) that affected biological pathways following treatment. Various assays were conducted to assess the impact of aging on vitality (lifespan and healthspan), mitochondrial health, and antioxidant capacity. Gene expression data were analyzed to reveal changes in longevity and stress-related pathways, and antioxidant capacity was evaluated based on protection against ROS. Mitochondrial health was measured by observing mitochondrial fragmentation in the aged animals.

## 2 Materials and methods

### 2.1 Botanical drugs, extract type and quality control

JadeAging is a proprietary Chinese botanical drug formulation composed of standardized dry extracts of three botanical drugs: Panax ginseng C.A.Mey [Araliaceae; Ginseng Radix et Rhizoma], Rehmannia glutinosa Libosch [Scrophulariaceae; Rehmanniae Radix], and Poria cocos (Schw.) Wolf [Polyporaceae; Poria]. All three botanical drugs are covered by monographs in the Chinese Pharmacopoeia. For brevity, these botanical drugs are hereafter referred to as rehmannia, poria, and ginseng, respectively.

Standardized dry extracts of each botanical drug and the final JadeAging formulation were manufactured under Good Manufacturing Practice (GMP) conditions by Qingdao Chenland Biotechnology Co., Ltd. (Qingdao, China). The identity of the botanical drugs was authenticated by the manufacturer’s quality control department using macroscopic and microscopic examination in accordance with the respective pharmacopoeial monographs. Batch numbers, drug–extract ratios, extraction solvents, contents of key marker metabolites, and other pharmaceutical quality parameters are summarized in the certificates of analysis provided by the manufacturer (Supplementary Material). All botanical materials were obtained from cultivated sources that are not listed under the CITES appendices, and collection and processing complied with national regulations and the principles of the Nagoya Protocol on Access and Benefit-Sharing (Buck and Hamilton, 2011).

Chemical characterisation of the three botanical drug extracts and of JadeAging followed the recommendations of the ConPhyMP statement (Heinrich et al., 2022). In brief, reverse-phase HPLC–DAD fingerprinting was used for all extracts, and representative marker metabolites were quantified to define the chemical profile and to assess batch-to-batch consistency. Details of the analytical procedures, chromatographic conditions, fingerprint chromatograms, and similarity analyses are described in the Supplementary Methods and shown in Supplementary Material. Reporting of the botanical drugs and extracts in this study adheres to the GA-online best-practice checklist.

### 2.2 Animal maintenance and media

All assays were performed in the nematode *C. elegans*. To prevent chemical modification or metabolism of the test article by the food bacteria, animals were fed on a lawn of inactivated *Escherichia coli*, strain OP50. OP50 cultures were inactivated by exposure to 0.5% paraformaldehyde for 1 h followed by washing five times with M9 (Beydoun et al., 2021). Bacteria were dispersed by passing through a 5-µm filter during the wash steps. The quantity and distribution of food bacteria were calibrated to ensure adequate access to food for the duration of the assay, while maintaining the visibility of the animals.

### 2.3 Compound delivery

For all phenotypic assays, standardized dry extracts of rehmannia, poria, ginseng, and JadeAging were used as test substances. Stock solutions were prepared based on preliminary solubility tests. Rehmannia and ginseng extracts were readily soluble in water and were therefore dissolved in sterile distilled water. Poria and JadeAging extracts showed limited aqueous solubility and were dissolved in 10% (v/v) dimethyl sulfoxide (DMSO) to obtain concentrated stock solutions. All stocks were dispersed by sonication and sterilized by filtration through 0.2-µm membranes.

Immediately before each experiment, stock solutions were diluted with assay medium to the desired working concentrations (Tables 2 and 3). The final DMSO concentration in all treatments, including vehicle controls, was adjusted to 0.1% (v/v). Previous work has shown that DMSO concentrations up to 0.5% have little or no effect on lifespan, development, fertility, or locomotion in *C. elegans*, and 0.1% DMSO is widely used as a well-tolerated vehicle in *C. elegans* toxicology and aging assays (AlOkda and van Raamsdonk, 2022).

### 2.4 Growth and development

The ideal dose of a compound or formulation must optimize its effectiveness while minimizing its adverse effects. Because *C. elegans* physiology differs from that of humans, cultured cells, and other animal models, we conducted a series of experiments to empirically determine the ideal dosage and delivery strategy for treatment with JadeAging, the main components of which are: Rehmannia root (*R. glutinosa*, hereafter referred to as rehmannia), *Poria sclerotium* (*P. cocos*, hereafter referred to as poria), and Asian ginseng rhizome (*P. ginseng*, hereafter referred to as ginseng).

#### 2.4.1 Solubility and compound delivery

Solubilization stock solutions were prepared for each compound and are listed in (Table 1). All compounds were dissolved in the solvent, dispersed by sonication, then sterilized by passing through a 0.2-µm filter. The stock solutions were then diluted to the desired concentration for each assay.

**TABLE 1:** Stock preparations of compounds.

Compound	Solvent	Stock concentration(mg/mL)
Rehmannia	Water	150
Poria	10% DMSO	100
Ginseng	Water	100
JadeAging	10% DMSO	100

Delivery strategy: The indicated compound dosage for each assay was based on the total volume of the delivery medium. The compound stock was diluted in a working solution and combined directly with food before seeding onto agar plates. The food spots were dried slowly, allowing the compound to diffuse into the food bacteria and agar for at least 24 h before the animals were introduced. Delivery into animals by absorption was facilitated using 0.1% DMSO as a vehicle.

#### 2.4.2 Growth and development assay

High-resolution imaging and automated detection were used to precisely measure the growth rate of the animals from hatching to the first d of adulthood. The *C. elegans* growth and development assay is highly sensitive to chemical and nutritional perturbations and is widely used in toxicology studies. Performing this test over a range of doses helps to identify a set of doses that have a physiological impact and exclude dose ranges that are likely too toxic to benefit the animal over the course of its lifespan.

Rehmannia, poria, and ginseng were tested at seven concentrations chosen based on concentrations previously published in the scientific literature to determine their effect on growth (Table 2). The size of the animals grown on these compounds was compared to that of animals grown on food without additives (vehicle control or VC). Working concentrations were calculated from the stock solutions based on the desired final doses, and the values reported in Table 2 were rounded to the nearest integer (µg/mL) for clarity of presentation.

**TABLE 2:** Concentrations tested for effects on growth and development.

Rehmannia	Poria	Ginseng
6 mg/mL	1 mg/mL	4 mg/mL
2 mg/mL	333 μg/mL	1.3 mg/mL
667 μg/mL	111 μg/mL	444 μg/mL
222 μg/mL	37 μg/mL	148 μg/mL
74 μg/mL	12 μg/mL	49 μg/mL
25 μg/mL	4 μg/mL	16 μg/mL
8 μg/mL	1 μg/mL	5 μg/mL

^*^ Data are shown as mean ± SD (n = 3). All concentrations are reported after rounding to the nearest integer (µg/mL).

### 2.5 Vitality platform

Instrumentation: To obtain high-resolution lifespan data and eliminate confounding factors such as worm handling and operator bias, lifespan data were collected using an automated Life and Death Instrument (LaDI), adapted from an Automated Lifespan Machine (ALM) (Stroustrup et al., 2013). LaDI contains proprietary modififications to improve temperature stability and image acquisition. The unit consists of a modifified EPSON V850 or V800 scanner and images are processed and analyzed using the ALM software (Stroustrup et al., 2013). The machine time-of-death calls are trained and validated using the “storyboarding” feature of the ALM software.

Procedures: Vitality measurements were adapted from published protocols (Lucanic et al., 2017). Animal populations were expanded and synchronized by bleaching, allowing the larvae to hatch and arrest in a food-deprived state. Asdevelopment and aging are temperature-dependent, the animals were kept strictly at 20 ° ± 1 °C throughout the experiment in a temperature-controlled room equipped with multiple temperature sensors. To eliminate the effects of bacterial metabolism and growth on the lifespan, synchronized animals were exposed only to inactivated bacterial food. To suppress progeny formation, animals were transferred to media containing 5-Fluorodeoxyuridine (FUdR) within 54–60 h post-plating. Animals were inspected 24 and 48 h after this transfer to confirm infertility. Finally, the animals were inspected for general health and morphology before being transferred to freshly prepared plates for the remainder of the assay. The animals were incubated for an additional 2 days and inspected again before loading onto the LaDI. Images of the animals were collected for 35 days with no interruptions or manipulations.

Some plates were excluded after pre-defined technical quality checks; however, plate numbers and worm counts for all replicates still exceeded the minimum required for statistical significance. The total number of lifespans recorded per condition surpassed the 150 animals required to eliminate subsampling errors and detect lifespan differences of approximately 10% (Gruber et al., 2009). Plates were excluded only when clear technical problems were present (e.g., microbial contamination, severe agar drying or cracking, or scanner and image-acquisition failure), and these exclusions were made in a treatment-blind fashion before any statistical analysis. No individual animals were removed based on their survival time, and all animals on plates that passed quality control were included in the analysis.

To assess potential confounding by non-treatment factors, we additionally fitted Cox proportional hazards models using the Lifelines software package was entered as the main exposure, and plate ID, experimental batch, and scanner position were included as covariates. None of these non-treatment covariates had a significant effect on the hazard of death (P > 0.05), and their inclusion did not materially change the treatment hazard ratios, indicating that factors other than treatment did not measurably confound the survival data. Qualitatively, animals on all included plates appeared morphologically and behaviorally normal, further suggesting that they were not affected by confounding hazards such as contamination or overt toxicity.

A minimum of 250 animals was used for each condition on the Vitality Platform. The fifinal number of animals tested in each compound group are as follows: VC, 336; rehmannia: 304; poria, 336; ginseng: 275; and JadeAging, 347.

### 2.6 Lifespan analysis

The time of death calls exported from the ALM software were analyzed and plotted using the Lifelines software package (lifelines, survival analysis in Python). Additional analysis was performed using OASIS2 analysis software (Han et al., 2016). A standard Mantel-Cox log-rank test with Holm-Bonferroni correction for multiple comparisons was used to compare the survival curves over the course of the lifespan by, assigning equal weights to each time point.

### 2.7 Healthspan analysis

Worm movement was tracked from images acquired by LaDI during the lifespan assay. Movement features were extracted and analyzed using custom software. Worm activity and movement serves as a proxy for animal health. State transitions were assessed using a Hidden Markov Model (HMM) (Oswal et al., 2022) trained on N2 wild-type *C. elegans* at 20 °C. The HMM uses the features of the animal’s movement to determine the life state of the worm. The transition from active crawling on a plate (fast/vigorous movement) to the slow-moving phase is the age of the last vigorous movement. Gross curation was performed by manual analysis of the worms and worm deaths.

### 2.8 Antioxidant capacity platform

A large population of wild-type *C. elegans* was synchronized and grown to the L4 juvenile stage. Liquid culture medium was prepared fresh as follows: S medium, 50 μM FUdR, 200 μg/mL Streptomycin, and filtered inactivated OP50. The L4 animals were resuspended in liquid culture at approximately two animals/μL. Liquid culture mediuma (80 µL) containing animals were added to each well of a 96-well microplate. Compound (10 µL) or vehicle were added to each well to reach the desired compound concentration. Microplates were sealed and incubated at 20 °C overnight on a shaker. The following day, the activity was measured using the wMicrotracker (PhylumTech) for 2 h. This is the basal measurement at which the activity is normalized. Next, 10 µL of Paraquat was pipetted to each well for a fifinal concentration of 10 mM. Activity wasrecorded on a wMicrotracker for 24 h. Activity was grouped into blocks of 2 h, and the percent activity was calculated by normalizing to the basal activity measurements. The data shown are based on three biological replicates consisting of four (JadeAging, rehmannia, poria, and ginseng treatments) or eight (positive and negative control) technical replicates.

### 2.9 Mitochondrial function platform

Microscopy: All slides were prepared immediately before imaging, and live animals were imaged within 1 h of mounting. Animals were transferred by picking to a 10 µL cold M9 buffer on an adhesion-coated glass slide. Thirty microliters of 30% Pluronic gel (NemaGel, InVivo Biosystems) was added to M9 and compressed with a cold #1.5 coverglass. The slide was incubated for 2 min at 25 °C to harden the NemaGel, then imaged immediately. Images were acquired using a Nikon Eclipse Ti2 Spinning Disk Confocal Microscope equipped with a ×40 immersion objective lens. The laser intensity, exposure, and gain settings were maintained constant for all the images collected. Head and tail images were collected from each worm from regions between and excluding the pharynx, tail tip, and vulva. The images were processed to create a maximum-intensity projection.

Manual image analysis: All images were blinded before being delivered to a group of six viewers. Viewers were instructed to rate mitochondrial fragmentation from 0 (completely intact) to 3 (completely disrupted) for each image. The scores from each viewer were averaged for each image. Statistical analysis was performed using analysis of variance (ANOVA) and a *post hoc* t-test using custom code in R.

### 2.10 Genetic signature platform

#### 2.10.1 Worm harvesting and RNA extraction

Adult animals were harvested, cleaned by fifiltration, and frozen in RNA stabilization reagent. RNA was extracted from all samples using a Quick-RNA Miniprep Plus Kit (Zymo Research). All samples exceeded our threshold for RNA quantity and quality. RNA samples were submitted to Novogene Co. Ltd., subjected to more stringent QC, tested on a Qubit for concentration and run on an agarose gel and on an Agilent 2100 to assess RNA quality and integrity. During Novogene’s QC, several samples received scores below the standard quality limits. After discussion with Novogene we proceed with the samples with an RIN of 4.3 and above.

#### 2.10.2 RNA-seq library preparation and sequencing

Total RNA was enriched for poly mRNA using oligo(dT) paramagnetic beads. DNA libraries were constructed from the input mRNA using a NEBNext UltraTM II RNA Library Prep Kit. This creates a ready-to-sequence dsDNA library that retains the strand-specific information of the original mRNA. These libraries were further tested using a Qubit for concentration and Agilent 2100 for library size distribution and quality. To properly pool the libraries and load them onto sequencing lanes to ensure the correct number of reads per sample, an even more precise quantification of the library was done *via* qPCR, and the samples were loaded onto the NovaSeq 6000 platform for a paired-end sequencing run of 150 bp for each end (PE150). Loading concentrations were designed to obtain at least 6.0 Gb (the number of billion bases of raw data, determined by the number of reads multiplied by the length of each read). Quality control of the sequencing run data was performed internally using Novogene. The raw dataset was analyzed as follows.

1. The distribution of base quality along the length of the sequencing read.
2. The distribution of error rate along the length of the sequencing read.
3. The distribution of A/T/G/C bases along the length of the sequencing read.
4. The distribution of raw data filtering results based on the following three criteria:
  - Removing reads containing adapter sequence.
  - Remove reads with N > 10% (where N means “base cannot be determined”).
  - Remove reads with a low quality (Qscore ≤5) for ≥50% of its total bases.

**FIGURE 1:**
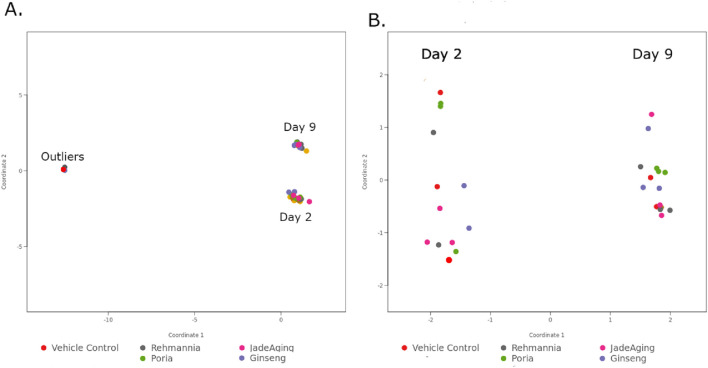
PCA plots reduced data sets two dimensions (2D) to reveal patterns in complex data sets. When the data is plotted along these two dimensions, the samples form clusters based on their overall similarity to one another. **(A)** Shows the outliers clustering as a separate group from the rest of the days 2 and 9 samples. **(B)** After eliminating the outlier samples, the days 2 and 9 samples form distinct clusters separated horizontally.

As a QC step we used a principal component analysis (PCA) for all samples and conditions. Examining this PCA revealed that three samples were not behaving as expected Figure 1A. We eliminated these three samples from our expression analysis and reran the PCA (Figure 1B). With this restricted set of data, all day 2 treatments were horizontally separated from all day 9 treatments along the first principal component axis (PC1), which is what we expect from samples collected at different stages in the life cycle. Other QC metrics, such as the distribution of the log of unnormalized gene counts and the distribution of mapped reads over gene features across the genome, were also evaluated. All QC metrics were met for the restricted datasets.

#### 2.10.3 Gene expression analysis

##### 2.10.3.1 ROSALIND® RNA-seq methods

Data was analyzed using ROSALIND® (https://rosalind.bio/), with a HyperScale architecture developed by ROSALIND, Inc. (San Diego, CA). Individual sample counts were normalized to Relative Log Expression (RLE) using the DESeq2 R library (Love et al., 2014). Read Distribution percentages, violin plots, identity heatmaps, and sample MDS plots were generated as part of the QC step. DEseq2 was also used to calculate fold changes and p-values and to perform optional covariate correction. Clustering of genes for the final DEG heatmap of DEGs was performed using the partitioning around medoids) method with the fpc R library (Hennig, C. Cran-package fpc. https://cran.r-project.org/web/packages/fpc/index.html).

Differential gene expression was assessed using EdgeR (Robinson et al., 2010), with false likelihood ratio tests based on linear models. Likelihood ratios were used to determine p-values which were subsequently corrected for using the BH false discovery rate (fdr) method.

#### 2.10.4 Genetic pathway analysis

Gene Ontology (GO) enrichment analysis on DEGs was conducted using GeneOntology.org and ROSALIND. For ROSALIND analysis, a hypergeometric distribution was used to analyze the enrichment of pathways, gene ontology, domain structures, and other ontologies. The topGO R library (Alexa and Rahnenführer, n. d.) was used to determine the local similarities and dependencies between GO terms to perform ELIM pruning correction. Several databases were referenced for enrichment analysis, including Interpro (Mi et al., 2019),NCBI (Geer et al., 2010), MSigDB (Subramanian et al., 2005; Liberzon et al., 2011), REACTOME (Gillespie et al., 2022), WikiPathways (Slenter et al., 2018). Enrichment was calculated relative to a set of background genes relevant to the experiments.

#### 2.10.5 Targeted pathway analysis

*Caenorhabditis elegans* genes orthologous to human genes and pathways of interest were identified using OrthoList2 (Kim et al., 2018). The identified *C. elegans* genes were extracted from a set of DEGs for focussed analysis. Gene components of the longevity pathway were determined using WormBase (Davis et al., 2022), Kyoto Encyclopedia of Genes and Genomes (KEGG) (Kanehisa and Sato, 2020), and REACTOME. To identify other pathways enriched in the gene expression data, the set of DEG was mapped to the KEGG pathway database using the KEGG Mapper (Kanehisa and Sato, 2020).

## 3 Results

### 3.1 Growth and development assay results

**FIGURE 2:**
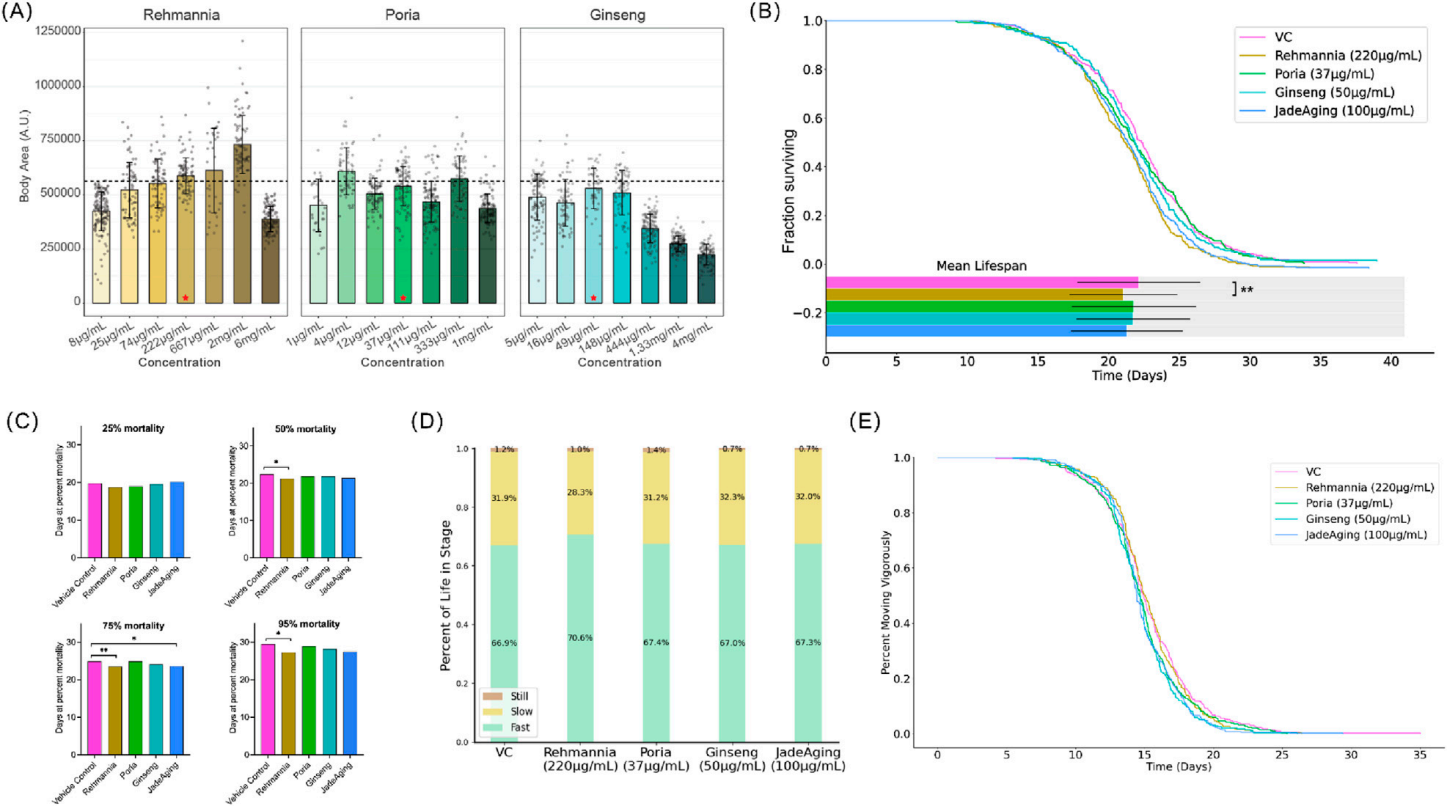
**(A)** Animal size at adulthood. Average animal size at adulthood (4 days post-hatching) was determined from high-resolution imaging and automated detection and tracking software (WormLab). The dashed line indicates the average size of untreated animals (VC). Size was quantified as area. The box shows the mean area and dots are individual animals. Error bars represent standard error of the mean (SEM). Red stars indicate selected concentrations for further study. **(B)** Lifespan assay. Bars indicate the mean lifespan for each condition. Error bars represent the standard deviation. The means were compared using ANOVA followed by *post hoc* t-tests. *, P < 0.05; **, P < 0.01; ***, P < 0.001; ****, P < 0.0001. **(C)** Age at 25%, 50%, 75%, and 95% mortality. For each treatment group, the value shown at each mortality percentile represents the mean age (in days) at which 25%, 50%, 75% or 95% of the population had died, calculated from the same survival data as in **(A, B)**. Age at 50% mortality is equal to the median lifespan of the population. The corresponding statistical comparisons for age at 25%, 50%, 75% and 95% mortality are summarized in Table 5. **(D)** Activity phases over lifespan. Animals treated with rehmannia spent a large proportion of their lives in a fast-moving state. **(E)** Age at the last vigorous movement. Modeling was used to identify the time point at which each animal transitioned from vigorous to weak movements. The percentage of the population treated with each compound that moved vigorously daily is shown.

Compared to VC (dashed line, Figure 2A), only rehmannia showed an increase in growth. However, a noticeable increase in variance of size at the higher doses tested (Figure 2, brown bars) suggested that the optimal dose for testing rehmannia for effects on longevity would be below 667 μg/mL (Figure 1A). Poria did not cause concentration-dependent changes in size. Ginseng caused a decrease in size above 148 μg/mL, indicating that lower concentrations would be optimal for testing the effects of ginseng on longevity. In this assay, adult body area at day 1 serves as an integrative readout of developmental progression, nutritional status, and general wellbeing. Marked reductions in body area typically indicate growth retardation or systemic toxicity, whereas pronounced increases in body area can reflect abnormal fat accumulation or fluid retention rather than healthy growth. Therefore, we did not select the largest animals or the highest doses for downstream studies. Instead, for each compound we prioritized doses that produced adult body areas as close as possible to the vehicle control distribution, with minimal variance in size. On this basis, 222 μg/mL rehmannia, 37 μg/mL poria, and 50 μg/mL ginseng were chosen as working concentrations for chronic exposure in the Vitality Platform, and 100 μg/mL JadeAging was selected because it yielded adult body areas overlapping those of vehicle controls and showed no obvious signs of swelling, shrinkage, or morphological abnormality.

Based on the Growth and Development assays, appropriate compound concentrations were selected for each phenotyping platform, as described below. For the Vitality Platform, which involves chronic exposure over the entire adult lifespan, we selected, for each individual extract, the concentration that produced an adult body area distribution most similar to that of vehicle control animals and with minimal variance, thereby minimizing the risk of obesity-like enlargement or overt toxicity. This led to the selection of 222 μg/mL rehmannia, 37 μg/mL poria, and 50 μg/mL ginseng. In addition, 100 μg/mL JadeAging was selected for the Vitality Platform because it yielded adult body sizes comparable to those of vehicle controls without obvious swelling, shrinkage, or other morphological abnormalities.

The antioxidant platformwas run using three different concentrations of each compound. Because acute antioxidant effects are often observed at higher concentrations than those associated with lifespan extension, the lowest concentration roughly matched that used for the Vitality Platform, and two additional higher concentrations were selected, as shown in Table 3.

**TABLE 3:** Concentrations selected for testing antioxidative effects.

Rehmannia	Poria	Ginseng	JadeAging
220 μg/mL	33 μg/mL	56 μg/mL	111 μg/mL
670 μg/mL	100 μg/mL	167 μg/mL	333 μg/mL
2 mg/mL	300 μg/mL	500 μg/mL	1 mg/mL

The mitochondrial health platformwas run only for the JadeAging compound. Due to the chronic nature of exposure, such as with the Vitality Platform, a concentration of 100 μg/mL JadeAging was selected.

### 3.2 Vitality Platform

To determine whether JadeAging or its components affect health and longevity, we subjected *C. elegans* with the selected concentrations of each compound and assessed the lifespan of the population and activity levels over the course of the lifespan.

#### 3.2.1 Lifespan results

Animals were treated with a selected concentration of each compound and compared to untreated animals (vehicle control, VC). The treatment of animals with JadeAging or its components did not produce a detectable increase in lifespan (Figure 2B).

Lifespan data were represented as the proportion of animals surviving over time, known as the Kaplan-Meier Estimate of Survival function (Figure 2B). To determine whether any treatment group survived longer than the vehicle control group, survival curves were compared using a log-rank test (Table 4). Treatment with 220 μg/mL Rehmannia or 100 μg/mL JadeAging decreased lifespan when the curves were analyzed using the Mantel-Cox Log rank test corrected for multiple comparisons (Table 4). The mean days of death were compared using ANOVA followed by *post hoc* t-tests. The vehicle-treated control group exhibited a mean lifespan of 22.1 days. The mean population lifespan of animals treated with 220 μg/mL Rehmannia was significantly also lower than that of the vehicle control group (Figure 2B).

**TABLE 4:** Pairwise statistical analysis of survival curves.

	Mantel-Cox log rank	
Curve comparisons (vs. Vehicle control) (µg/mL)	Test statistic (Χ^2^)	P-value (Bonf. Corrected)
Rehmannia	15.85	**<0.001**
Poria	0.53	>0.05
Ginseng	2.71	>0.05
JadeAging	8.52	**<0.05**

The Mantel-Cox log-rank test is a non-parametric test that compares two survival functions across the duration of the lifespan. P-values were corrected for multiple comparisons using the Holm-Bonferroni method. Bold indicate statistical significance (P < 0.05).

**FIGURE 3:**
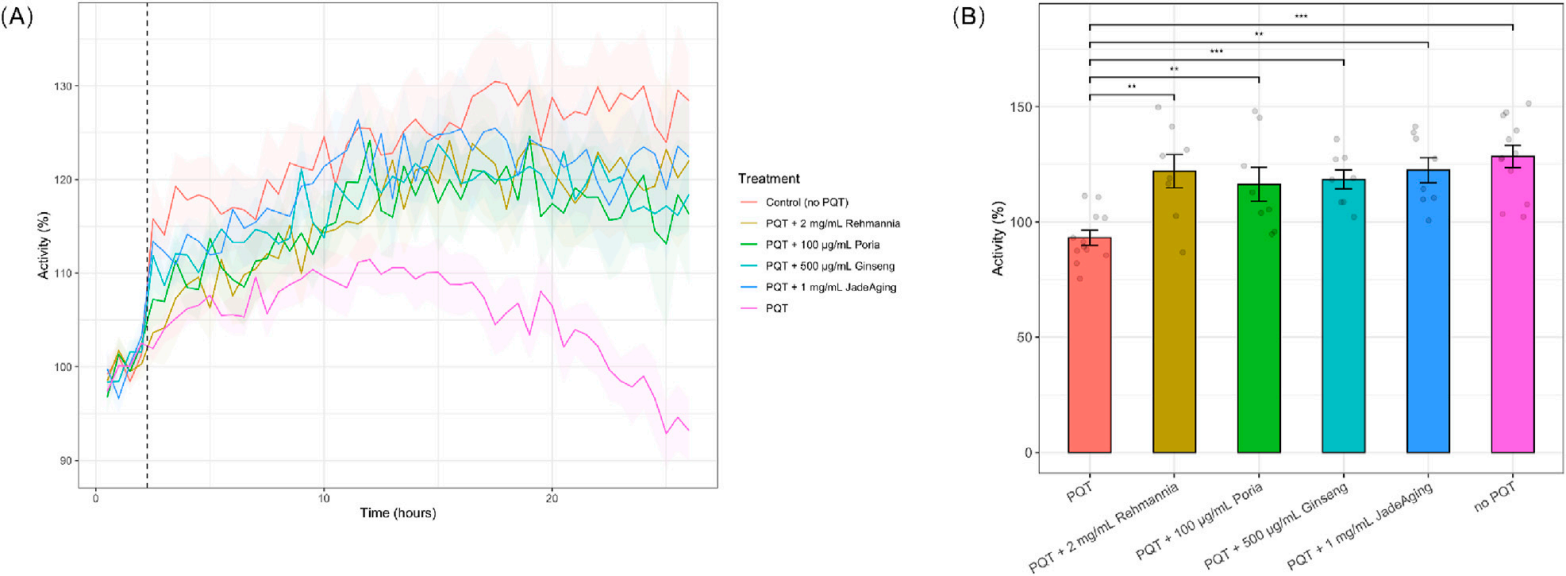
**(A)** Locomotion of animals after exposure to PQT. Animals were incubated overnight in JadeAging, one of its components, or vehicle control. Baseline activity of animals in each treatment group was recorded for 2 h prior to exposure to 10 mM PQT (dashed line). Activity was normalized to average baseline activity. Shading represents SEM. Plots are an average of two biological replicates. **(B)** Locomotion of animals after 24 h exposure to PQT. After 24 h of PQT exposure, animals that were pre-incubated with JadeAging or one of its components showed activity levels significantly higher than animals incubated with the vehicle control andf similarto those that had not been exposed to PQT. Error bars represent SEM. *, P < 0.05; **, P < 0.01; ***, P < 0.001; ****, P < 0.0001. Groups were compared using ANOVA followed by *post hoc* t-tests corrected for multiple comparisons (Bonferroni correction).

To further dissect how different phases of the lifespan were affected, we calculated, for each treatment group, the mean age at which 25%, 50%, 75% and 95% of the population had died (Figure 2C). These percentile-based mean ages were derived from the same survival curves shown in Figures 2A,B and provide a complementary summary of the average age at early-, mid- and late-life mortality. Overall, the mean ages at each mortality percentile closely mirrored the survival curves, and the few statistically significant differences observed were confined to late-life mortality, in agreement with the Fisher’s exact tests summarized in Table 5. These analyses are useful for examining early or late life-specific effects. Conventionally, the “maximum lifespan” is typically the 95th percentile of the lifespans recorded. The median lifespan (50% population survival) of the vehicle-treated control group was 22.3 days and the maximum lifespan was 29.5 days. Compared to the control group, the group treated with 220 μg/mL rehmannia had a significantly decreased median lifespan of 21.3 days (Table 5, P = 0.02) and a significantly decreased maximum lifespan of 27.2 days (Figure 3; Table 5, P = 0.02). Treatment with 100 μg/mL JadeAging produced a significant increase in late deaths at 75% mortality (Table 5, P = 0.04), but had no effect on the median or maximum lifespan (Table 5). None of the other treatment groups was significantly different from the vehicle control treated animals.

**TABLE 5:** Fisher’s exact test for survival differences at key time points.

Treatment (vs. Vehicle control) (µg/mL)	P-value at 25% mortality	P-value at 50% mortality	P-value at 75% mortality	P-value at 95% mortality
Rehmannia	>0.05	**<0.05**	**<0.01**	**<0.05**
Poria	>0.05	>0.05	>0.05	>0.05
Ginseng	>0.05	>0.05	>0.05	>0.05
JadeAging	>0.05	>0.05	**<0.05**	>0.05

Statistical analysis corresponding to the age at specific percent mortality shown in Figure 2.2. Age at 50% mortality is equal to the median. Bold indicate statistical significance (P < 0.05). P-values are corrected for multiple comparisons using the Holm-Bonferroni method.

Although several previous studies have shown the potential lifespan benefits of several components of JadeAging (Lee et al., 2007; Zeng et al., 2019; Yuan et al., 2019), our data surprisingly did not indicate that JadeAging or its components have a positive effect on lifespan at the concentrations tested.

#### 3.2.2 Healthspan results

To measure healthspan, the animals movements of the animals were quantified throughout their lifespan. Movement can indicate energy levels and health, as older animals typically exhibit decreased activity. Using our machine learning platform, each animal’s movement throughout its lifespan was classified as “Fast/Vigorous”, “Slow”, or “Still”, and the percentage of the lifespan each treatment group spent in these movement states is plotted below (Figure 2D). Animals treated with the 220 μg/mL rehmannia exhibited vigorous movement for a larger percentage of their lives (Figure 2D). The percentage of animals in a vigorous movement state throughout their lifespan was plotted for each treatment group (Figure 2E).

To determine whether any treatment group exhibited prolonged vigorous activity compared to the vehicle control group, the curves were compared using a log-rank test (Table 6). Both 50 μg/mL ginseng and 100 μg/mL JadeAging treatment groups were significantly different from vehicle control treated animals (Table 6), with an early increase in vigorous movement followed by a decrease (Figure 2E).

**TABLE 6:** Statistical analyses of healthspan curves.

	Mantel-Cox log rank	
Treatment comparison	Test statistic (Χ^2^)	P-value (Bonf. Corrected)
Rehmannia 220 μg/mL vs. vehicle contro	0.578	>0.05
Poria 37 μg/mL vs. vehicle control	3.101	>0.05
Ginseng 50 μg/mL vs. vehicle control	8.530	**<0.05**
JadeAging 100 μg/mL vs. vehicle control	8.273	**<0.05**

The Mantel-Cox log-rank test is a non-parametric test. P-values are corrected for multiple comparisons using the Holm-Bonferroni method. Bold indicate statistical significance (P < 0.05).

Collectively, these results indicate that treatment with JadeAging or its components did not significantly improve healthspan at the concentrations tested.

### 3.3 Antioxidant capacity platform results

ROS are produced by both normal cellular metabolism and exposure to environmental agents such as pollutants and radiation. Excess ROS can damage DNA, RNA, and proteins leading to cell death. Antioxidants are molecules that mitigate the damage caused by ROS. We tested whether JadeAging or its components mitigated the damage caused by exposure to a pesticide known to induce ROS formation.

ROS can lead to severe stress responses and ultimately, to the early death of animals. To explore the antioxidant capacity of JadeAging and its components, we recorded the locomotion of animals exposed to 10 mM paraquat (PQT), an organic compound often used as an herbicide that causes the release of ROS. Animals were preincubated with a vehicle control or one of the test compounds (rehmannia, poria, ginseng, or JadeAging) overnight. Although three concentrations of each compound were tested, only the concentration that best protected against ROS activity is shown in (Figure 3). The decrease in PQT activity observed after 10 h (Figure 3A) was likely due to the increasing effects of oxidative stress caused by prolonged exposure to PQT. Paraquat induces ROS generation, leading to cellular oxidative damage (Dilberger et al., 2019). Over time, this can result in diminished locomotor activity, as oxidative stress impairs the physiological functions of *C. elegans*.

After 24 h of exposure to PQT, locomotion was compared with that of animals that had not been exposed to PQT or had been exposed to PQT only, without incubation in JadeAging or one of its components. Treatment with each compound significantly protected against the effect of PQT (Figure 3A). Animals pre-incubated in JadeAging or one of its components before treatment with PQT exhibited the same activity levels as animals that had never been exposed to PQT (Figure 3B).

Treatment with JadeAging or any of its components resulted in greater activity under oxidative stress than the vehicle control, indicating that JadeAging and each of its components provides protection against oxidative stress. These data supported the ability of JadeAging to function as an antioxidant agent.

### 3.4 Mitochondrial health

Sarcopenia is the age-related loss of muscle strength. Muscle mitochondrial dysfunction is observed at the onset of sarcopenia, followed by subsequent changes in sarcomere structure, and ultimately, a decline in movement. We used a *C. elegans* strain with fluorescently labeled muscle cell nuclei and mitochondria to assess whether JadeAging ameliorates age-related dysfunction.

**FIGURE 4:**
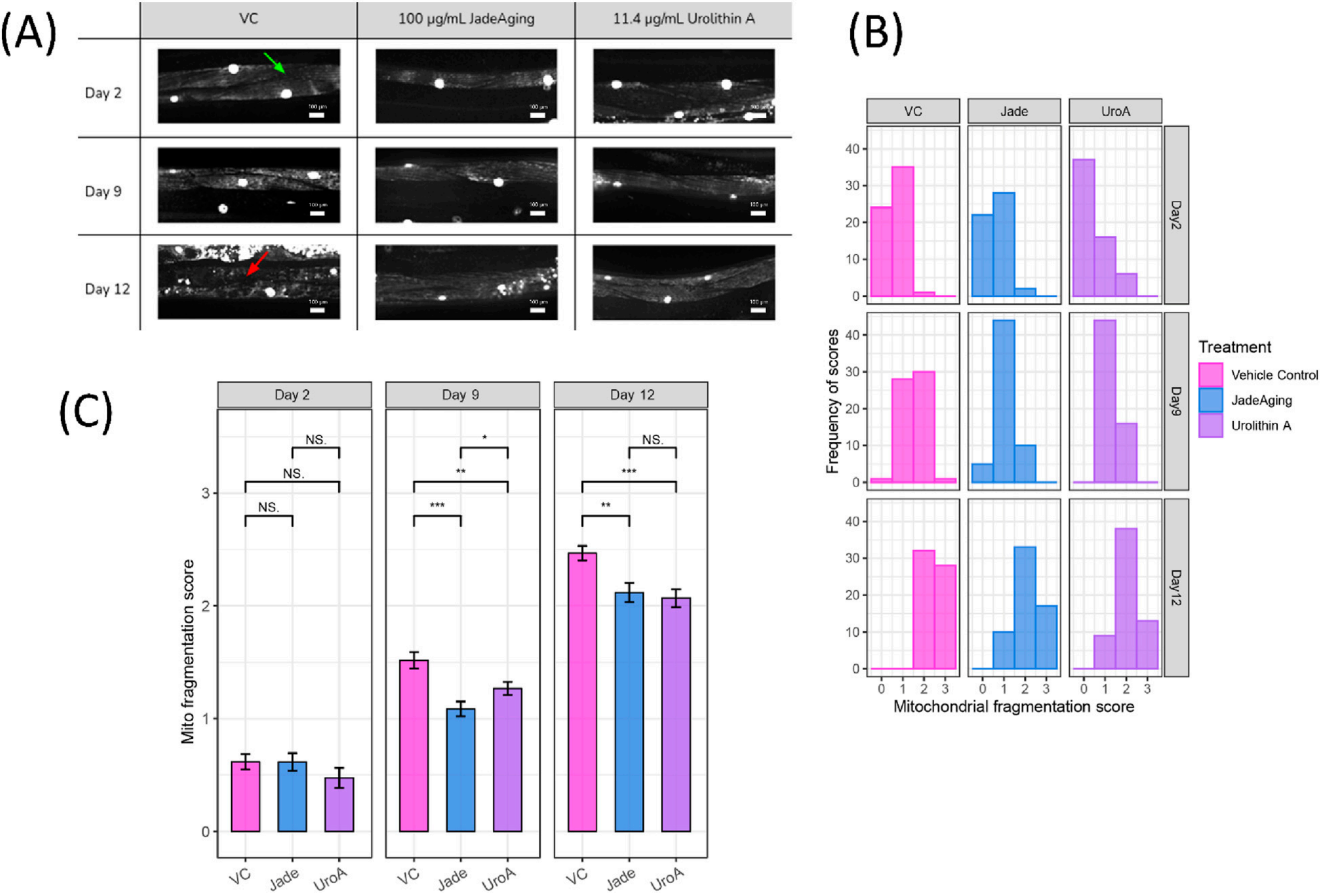
**(A)** Example images from each treatment group at each age. Example maximum intensity projection confocal images of muscles and mitochondria of animals on days 2, 9, and 12 of adulthood that have been treated with Vehicle control, JadeAging, or urolithin A. All images shown are from the “head” region between the pharynx and the vulva. Healthy muscle, indicated by a green arrow in the VC day 2 image, appears homogeneously striated. Fragmentation, indicated by a red arrow in the VC day 12 image, appears as non-uniform fluorescence. **(B)** Distribution of fragmentation scores in the treatment group at each age. Scores of 0–3 were assigned to each animal (n = 5 per group). Scores of 0 = completely intact, 1 = mild fragmentation, 2 = moderate fragmentation, and 3 = severe fragmentation **(C)** Average fragmentation score for the treatment group at each age. The average scores of animals from each group (N = 5) on each day showed significant protection from aging-related sarcopenia by JadeAging, as well as the positive control urolithin A. Error bars represent SEM. *, P < 0.05; **, P < 0.01; ***, P < 0.001; ****, P < 0.0001. Groups were compared using ANOVA followed by *post hoc* t-tests.

SD1347 animals, which have fluorescently labeled muscle cell nuclei and mitochondria, were placed on plates containing 100 μg/mL JadeAging, 11.4 μg/mL urolithin A, or vehicle control. Muscle images were obtained using a spinning disk confocal microscope on days 2, 9, and 12 of adulthood (Figure 4A).

All images were blinded for assessment by six viewers who assigned scores for each image as follows: 0 = completely intact, 1 = mild fragmentation, 2 = moderate fragmentation, and 3 = severe fragmentation. The distribution of the scores of images from each group (Figure 4B) as well as the average score of animals from each group (Figure 4C) were compared.

As expected, the distribution of the scores shifted to the right as the animals aged. However the shift in scores in animals treated with JadeAging or the positive control urolithin A less dramatic than that in animals treated with the vehicle control. On day 2 of adulthood, most animals in all the treatment groups have scores of either 0 or 1, indicating healthy mitochondria and muscles. On day 9, the fragmentation scores of animals treated with JadeAging were significantly lower than those of vehicle control treated animals (Figure 3C). On day 12, the animals were fairly old and exhibited substantial fragmentation. However, animals treated with JadeAging showed significantly less fragmentation than vehicle treated animals, and had a similar appearance to urolithin A treated animals (Figure 4C).

### 3.5 Genetic signature platform results

RNA-Seq was used to examine gene expression and determine the biological pathways affected by exposure to JadeAging and its components (rehmannia, poria, and ginseng). These pathways may explain the beneficial effects of JadeAging on health, including mitochondrial health and protection against ROS.

#### 3.5.1 Genetic pathway identification

To identify the potential mechanisms through which JadeAging affects health and vitality, global gene expression was analyzed usingmRNA-Seq. Both young (adult day 2) and aged (adult day 9) animals were collected after treatment with either 100 mg/mL JadeAging, 222 mg/mL rehmannia, 37 mg/mL poria, or 50 mg/mL ginseng. We identified the molecular pathways that were most likely modulated by treatment with JadeAging and gained insight into how these pathways contribute to the mechanism of action of JadeAging.

##### 3.5.1.1 Pathway mapping

**FIGURE 5:**
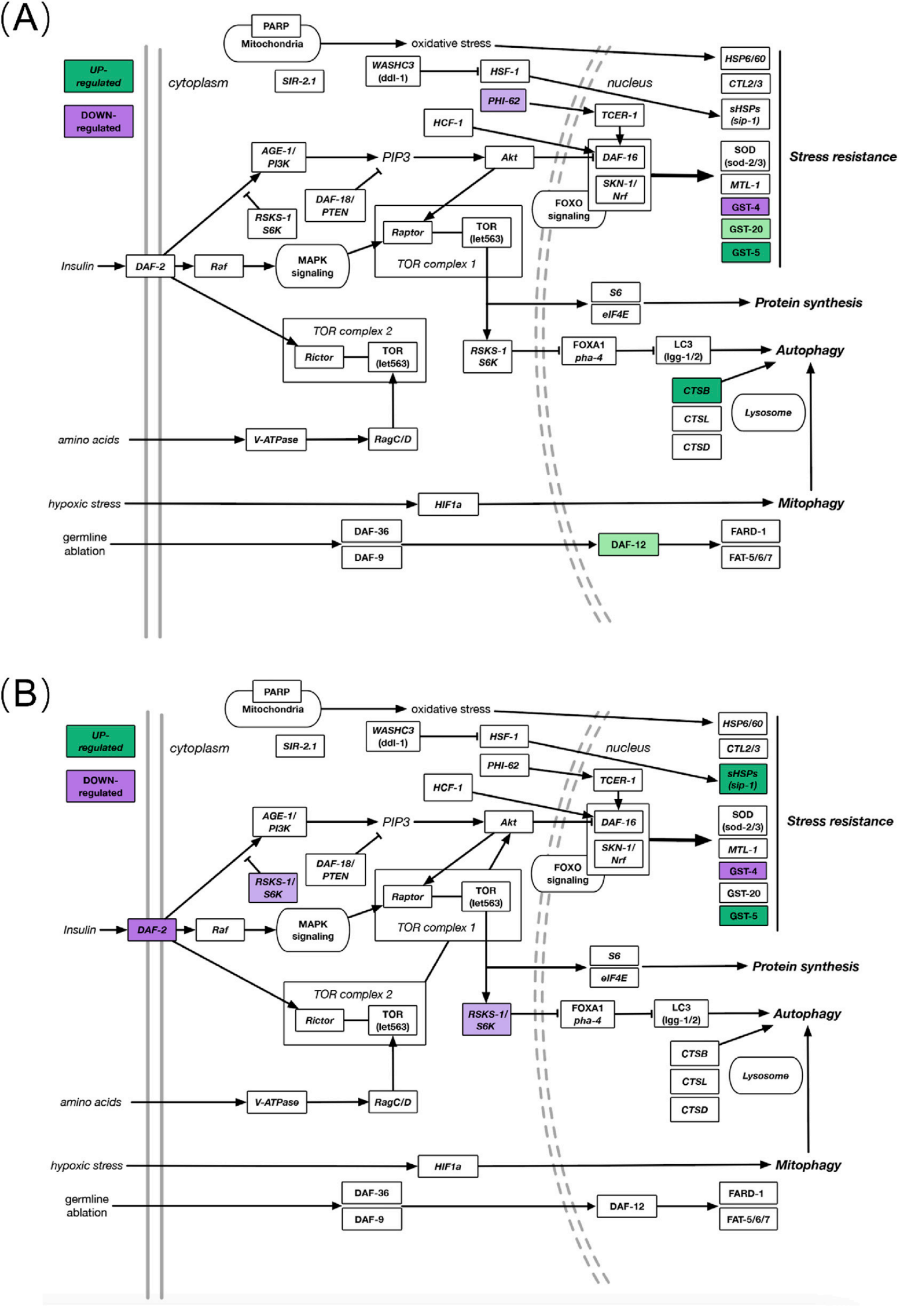
**(A)** DEGs in known aging-related gene pathways after JadeAging treatment in young (day 2) animals. **(B)** DEGs in known aging-related pathways after JadeAging treatment in aged (day 9) animals. Colored genes indicate the change in expression, >1.25-fold upregulated (green) or < −1.25-fold downregulated (purple). Pale colors, 0.05 < P < 0.1; dark colors, P < 0.05. Objects not colored indicate pathway components that were not differentially regulated in the dataset.

To place the transcriptomic data within the context of well-characterized biological pathways, we mapped how genes were affected by JadeAging within the canonical longevity pathway and additional supporting pathways (Figure 5).

Most gene expression products are proteins with specific functions. In this study, we used RNA-Seq technology to detect the effects of test substances on gene expression in *C. elegans*. Through literature review, we selected 93 genes related to anti-aging in *C. elegans* for analysis (in pathway analysis, we used a cutoff value of P < 0.1 and a fold change of ±1.25 to identify DEGs, and screened out the differential genes. Of the 93 genes known to be associated with longevity, we observed six that were differentially expressed in young adults (day 2) exposed to JadeAging and five that were differentially expressed in aged adults (day 9) after exposure to JadeAging (Figure 5; Table 7).

**TABLE 7:** Lifespan associated genes with altered expression in JadeAging vs. VC.

			Day 2		Day 9		
Genes	Human ortholog	Description	Fold change	*p*-val	Fold change	*p*-val	
C46G7.1	RNASEK/PHI-62	Uncharacterized protein	**−1.37**	0.06			Stress response
GST-4	GSTA1, GSTA2, GSTA3 GSTA4, HPGD5	Glutathione S-transferase 4	**−1.63**	6.24E-04	**−1.72**	2.35E-05	
GST-5	GSTA1, GSTA2, GSTA3 GSTA4, HPGD5	Putative glutathione S-transferase 5	**3.04**	<1E-10	**1.43**	0.01	
GST-20	GSTA1, GSTA2, GSTA3 GSTA4, HPGD5	Glutathione S-transferase	**1.29**	0.1			
SIP-1	Cryaa, CRYAB, HSPB1 HSPB2, HSPB3, HSPB6 HSPB8	Stress-induced protein 1			**1.3**	0.02	
RSKS-1	AKT1, AKT2, AKT3 RPS6KB1, RPS6KB2 SGK1, SGK2, SGK3	Ribosomal protein S6 kinase beta			**−1.34**	0.09	Autophagy /TOR
CPR-1	CTSB	Cathepsin B	**1.51**	9.02E-03			
DAF-2	IGF1R, INSR, INSRR	Insulin-like receptor subunit beta			**−1.48**	0.01	Insulin signaling
DAF-12	NR1H2, NR1H3, NR1H4 NR1I2, RARA, RARB RARG, THRB	Nuclear hormone receptor family member	**1.35**	0.08			Other

The bold values indicate statistically significant results (P < 0.05).

Several key members of thecanonical longevity pathway are differentially expressed in JadeAging-treated animals. Here we summarize the contribution of JadeAging to longevity in the context of these pathways.

###### 3.5.1.1.1 Nutrient sensing pathways that contribute to longevity

The insulin/insulin-like growth factor-1 (IIS) signaling pathway is the first pathway implicated in the genetic regulation of lifespan and aging. The discovery that the loss of function of the insulin receptor DAF-2 could more than double the lifespan of *C. elegans* was a landmark finding that helped launch the field of aging research (Kenyon et al., 1993). Daf-2 expression was downregulated in aged adult (day 9) animals treated with JadeAging (Figure 5B; Table 7).

Another important pathway involved in lifespan regulation through nutrient sensing activities is the Target of Rapamycin (TOR) pathway. TOR is a key nutrient sensor and a master regulator of growth and energy metabolism in animals (Blackwell et al., 2019). Signaling through TOR involves two distinct protein complexes, mTORC1 and mTORC2, which regulate different physiological processes that can affect the lifespan. mTORC1 acts primarily through the ribosomal S6 kinase (S6K). Inhibition of the S6K protein, encoded by rsks-1, significantly increases the lifespan (Hansen et al., 2007). Treatment with JadeAging caused a modest decrease in rsks-1 expression on day 9 animals (Figure 5B; Table 7). Interestingly, the IIS and TOR pathways interact, and animals with decreased daf-2 and rsks-1 function show synergistic lifespan extension (Chen et al., 2013).

###### 3.5.1.1.2 Stress-related pathways that contribute to longevity

Across species, from yeast to humans, moderate levels of stress evoke protective responses that promote longevity (Kishimoto et al., 2018). The transcription factor SKN-1 is an ortholog of human Nuclear Respiratory Factor (Nrf), which functions in conjunction with DAF-16 to activate transcriptional responses to xenobiotic and oxidative stress (Tullet et al., 2008). DAF-16/SKN-1 is best known as a key effector of insulin signaling but also as an interface with other longevity-promoting pathways. Although skn-1 and daf-16 expression remained unchanged, the downstream stress response effectors, glutathione transferases (GSTs), were affected. Gst-4 was downregulated in animals on days 2 and s 9 after JadeAging treatment (Figure 5; Table 7). In contrast, gst-5 was upregulated adults on days 2 and 9(Figure 5; Table 7) and gst-20 was modestly upregulated in adults on day 2 (Figure 5; Table 7). The gene neighboring effect is referred to as “expression piggybacking” (Ghanbarian and Hurst, 2015). This suggests that changes in the expression of one gene can influence the expression of its neighboring genes, especially when they are located within 100 kb of each other. This may be due to chromatin remodeling or the interaction of regulatory elements, leading to the co-regulation of neighboring genes. In *C. elegans*, following JadeAging treatment, the upregulation of GST-5 may influence the expression of nearby genes, such as GST-4, causing it to be downregulated due to chromatin dynamics or local regulatory factors. Gene interactions can alter protein folding, stability, and function. The three-dimensional (3D) structure of proteins is crucial for their biological activity and any changes can significantly affect their function (Manhart and Morozov, 2015). Changes in the protein structure can affect the interaction between JadeAging and its target proteins. When the conformation of a protein changes, JadeAging may not effectively bind to its target, thereby reducing its antioxidant, anti-aging, and other biological effects. However, minor conformational changes in the protein may not completely disrupt its binding ability, allowing it to maintain a certain level of pharmacological activity. Similar structural changes in proteins and their inhibitory effects on enzyme activity have been studied extensively (Kalhori et al., 2022). Studies have shown that changes in the secondary structure of proteins can significantly affect their catalytic activity, thereby influencing drug efficacy. This provides important theoretical support for understanding the effects of jadeaging on proteins. Decreases in gst-5 expression have been shown to reduce the lifespan (Ayyadevara et al., 2007), whereas other GSTs are likely to exert tissue-specific effects on longevity.

Another target of DAF-16 is the stress-response protein, SIP-1. Decreased sip-1 expression has been shown to decrease the lifespan (Morley and Morimoto, 2004). Sip-1 expression has been shown to be slightly elevated in animals lacking the rsks-1 gene as well (Seo et al., 2013). Day 9 adults treated with JadeAging showed upregulated sip-1 (Figure 5B; Table 7).Finally, germline ablation leads to lifespan extension and depends not only on DAF-16, but also on the nuclear hormone receptor, DAF-12. Increased DAF-12 levels increase the lifespan. Daf-12 was upregulated on day 2 in animals treated with JadeAging (Figure 5A; Table 7).

###### 3.5.1.1.3 Interaction of JadeAging components

We also examined longevity-related DEGs in animals treated with the three components of JadeAging: rehmannia, poria, and ginseng. Some genes that were upregulated in JadeAging, such as cpr-1, gst-5, and gst-20, were also upregulated in ginseng-treated animals. Similarly, gst-4, which was downregulated in JadeAging, was also downregulated in poria and rehmannia treated animals. In contrast, some genes that were differentially expressed in JadeAging treated animals, such as rsks-1, sip-1, phi-62, daf-12, and daf-2, were not significantly differentially expressed in any of the components of JadeAging.

##### 3.5.1.2 GO enrichment

In transcriptomic analysis, examining the coordinated regulation of genes grouped according to their functions can reveal changes in biological processes that are not apparent at the individual genes levels. GO enrichment analysis is a common approach that groups DEGs based on their annotated molecular functions. For this analysis a more conventional significance cutoff of P < 0.05 and fold change cutoff of ±1.5 were used. JadeAging treatment showed no significant enrichment of GO terms on day 9, and poria showed no enrichment on eitherday 2 or 9. The GO term enrichment for JadeAging, ginseng, and rehmannia treatments on day 2 is presentedin Tables 8–10.

**TABLE 8:** Top enriched biological process GO terms JadeAging vs. control day 2.

GO ID	Term	N	UP	Down	FDR-adjusted P-value	Elim pruning P-value
GO:0045087	Innate immune response	239	6	3	1.80E-05	6.30E-09
GO:0042738	Exogenous drug catabolic process	39	5	0	9.50E-05	6.00E-08
GO:0006805	Xenobiotic metabolic process	48	5	0	0.00018	1.80E-07
GO:0055114	Oxidation-reduction process	632	9	0	0.01284	2.00E-05
GO:0050829	Defense response to gram-negative bacterium	97	3	1	0.04941	0.00012

**TABLE 9:** Top enriched biological process GO terms ginseng vs. control day 2.

GO ID	Term	N	UP	Down	FDR-adjusted P-value	Elim pruning P-value
GO:0045087	Innate immune response	239	24	1	2.70E-09	<1E-10
GO:0055114	Oxidation-reduction process	632	35	5	2.70E-09	<1E-10
GO:0042738	Exogenous drug catabolic process	39	7	2	3.60E-05	3.40E-08
GO:0006805	Xenobiotic metabolic process	48	7	2	1.60E-04	2.30E-07
GO:0006629	Lipid metabolic process	473	24	0	1.96E-03	3.22E-03
GO:0009607	Response to biotic stimulus	196	12	2	6.12E-03	3.17E-02
GO:0043207	Response to external biotic stimulus	196	12	2	6.12E-03	3.17E-02
GO:0051707	Response to other organism	196	12	2	6.12E-03	3.17E-02
GO:0050829	Defense response to gram-negative bacterium	97	8	1	2.14E-02	6.40E-05
GO:0097501	Stress response to metal ion	26	5	0	3.35E-02	2.73E-02

**TABLE 10:** Top enriched biological process GO terms rehmannia vs. control day 2.

GO ID	Term	N	UP	Down	FDR-adjusted P-value	Elim pruning P-value
GO:0045087	Innate immune response	239	4	13	3.60E-09	<1E-10
GO:0050830	Defense response to gram-positive bacterium	70	2	6	2.50E-05	4.80E-08
GO:0050829	Defense response to gram-negative bacterium	97	2	4	4.02E-02	8.30E-05

JadeAging, ginseng, and rahmannia treated samples showed enrichment for GO terms related to defense against Gram-negative bacteria and innate immune response. Activation of the innate immune response is associated with increased lifespan (Kumar et al., 2019). Both JadeAging and ginseng treated samples also showed enrichment for GO terms likely related to detoxification and metabolism of the treatment compounds, exogenous drug catabolic process, and xenobiotic metabolic process. In addition, both were enriched in the oxidation-reduction process GO term, which supports the oxidative stress resistance observed in this study.

##### 3.5.1.3 Differential gene expression

**FIGURE 6:**
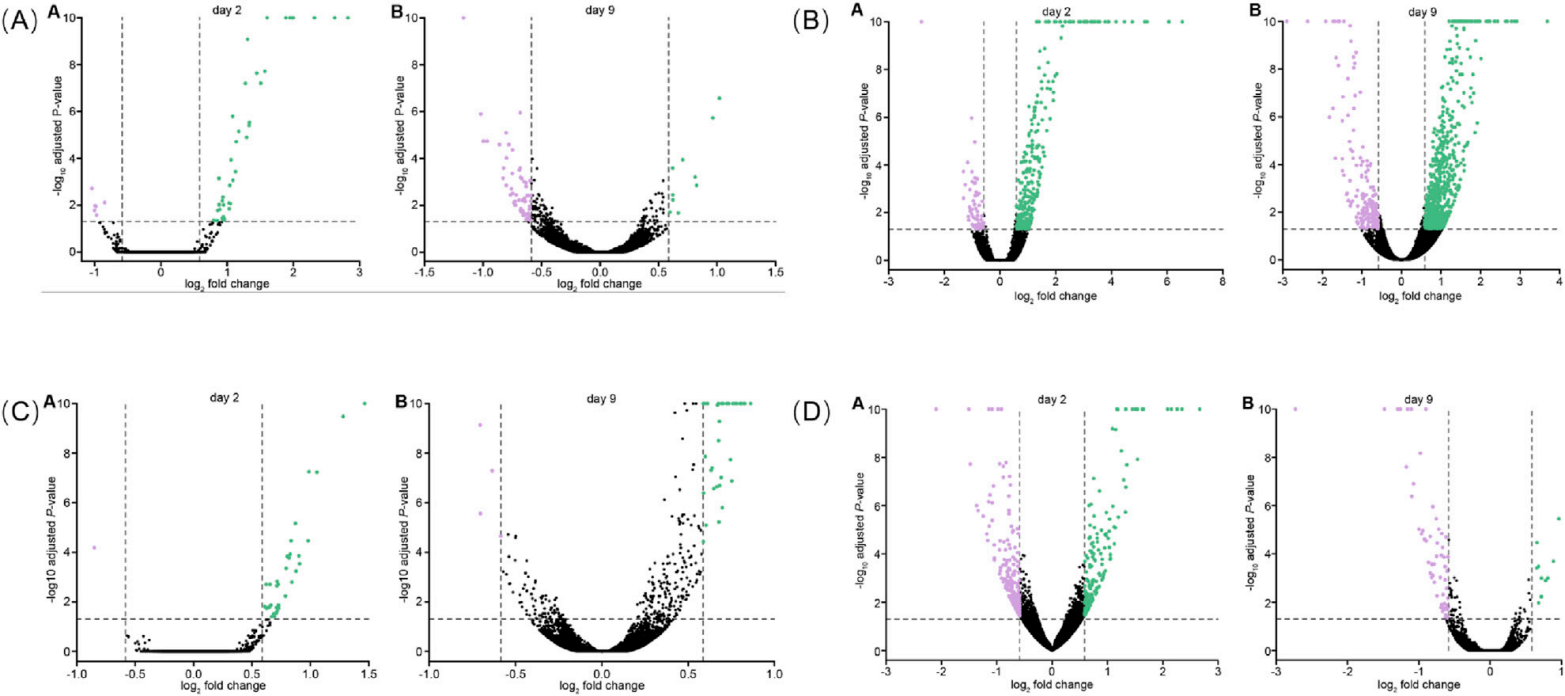
**(A)** Volcano plot of DEGs for JadeAging on days 2 A and 9 B. **(B)** Volcano plot of DEGs for ginseng on days 2 A and B **(C)** Volcano plot of DEGs for poria on days 2 A and B. **(D)** Volcano plot of DEGss for rehmannia on days 2 A 9 B. Each dot represents a gene used for comparison. The log ratio of the fold change is on the X-axis, and the negative log of the P-adj/P-value is on the Y-axis. Colored dots indicate differential gene expression exceeding the defined significance and fold-change thresholds. Black dots = not significant, green dots = > 1.5-fold change in expression and P-value <0.05, purple = < −1.5-fold change in expression and P-value <0.05.

To identify genes that were differentially expressed with JadeAging treatment in an unbiased manner, the gene counts for each JadeAging-treated samples were compared with those of the vehicle control. DEGs were defined as genes whose expression levels exceededs 1.5-fold, with an FDR-adjusted P-value of <0.05. However, a continuum of genes exists above and below these thresholds. The genetic response to JadeAging treatment was illustrated using volcano plots (Figure 6A). Volcano plots are a type of scatterplot in which the statistical significance and magnitude of change are plotted against each gene.

Volcano plots for ginseng (Figure 6B), poria (Figure 6C), and rehmannia (Figure 6D) are presented below.

**FIGURE 7:**
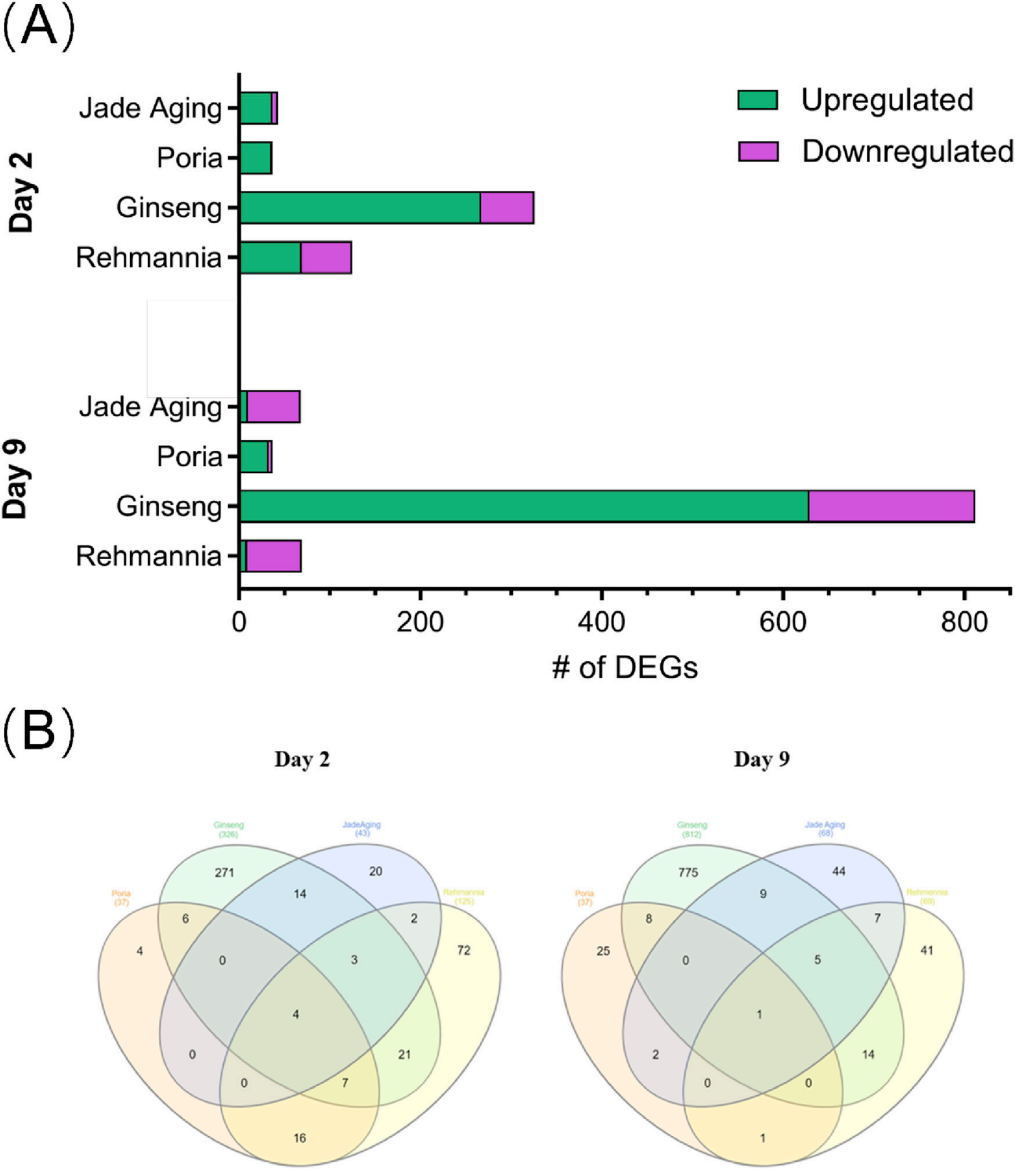
**(A)** The number of DEGs for each treatment group. **(B)** Venn diagrams showing the overlap in DEGs between treatment groups on day 2 (left) and day 9 (right).

We identified DEGs in the samples from days 2 and 9 in all treatment groups (Figure 7A). Ginseng-treated animals showed the largest number of DEGs on days 2 (326 DEGs) and 9 (812 DEGs). We also characterized the overlap of DEGs among the four treatment groups on days 2 and 9 using Venn diagrams (Heberle et al., 2015) (Figure 7B). The Venn analysis therefore highlighted five key genes—pgp-1, gba-2, cyp-13A5, oac-14 and best-1—that were commonly modulated across all four treatments at one or both time points (Figure 7B). This specific transcriptional signature suggests a coordinated mechanism of action. Pgp-1, an ABC transporter homolog, has been implicated in xenobiotic resistance and lipid homeostasis, and may contribute to the reduced accumulation of toxic metabolites in treated animals. The simultaneous modulation of gba-2 and best-1 (a chloride channel) points to a coordinated impact on lysosomal function and cellular ion balance, processes that are tightly linked to aging and stress resistance. Furthermore, cyp-13A5 (a cytochrome P450 enzyme) and oac-14 (an organic anion transporter) likely reflect adaptive activation of metabolic detoxification pathways. We propose that the concerted regulation of these genes underpins at least part of JadeAging’s ability to enhance healthspan by promoting cellular resilience and metabolic homeostasis.

JadeAging and ginseng had the most DEGs with 21 on day 2 and 15 on day 9, respectively. On day 2, four genes were identified as DEGs in all four treatment groups: pgp-1, gba-2, cyp-13A5, and oac-14. On day 9, only one gene was identified as a DEG in all four treatment groups: best-1. All genes that were reported as differentially expressed for JadeAging *versus* vehicle control at day 2 and day 9 in the original RNA-seq analysis report, together with their fold changes and FDR-adjusted P-values, have been compiled in Supplementary Table S1 (DEG sheet) and are sorted by the absolute value of the fold change within each comparison. The overlap of DEGs between JadeAging and the individual component extracts at day 2 and day 9 is reproduced in the Overlap sheet of Supplementary Table S1.

## 4 Discussion

The primary focus of geroscience, which constitutes the main scientific endeavor in human aging, is the hypothesis that aging is the primary cause of numerous chronic late-life diseases, including Alzheimer’s disease, chronic kidney disease, coronary artery disease (CAD), osteoarthritis, stroke, type 2 diabetes mellitus (T2DM), and prevalent cancers such as breast, prostate, and colorectal cancer (Kennedy et al., 2014). Various experimental interventions in laboratory models have demonstrated that decelerating the aging process may simultaneously prevent many diseases by slowing and partially reversing aging characteristics (Melzer et al., 2020).

This study suggests that JadeAging delays the aging process through multiple mechanisms. Similar to how natural compounds like Rebeccamycin are thought to exert anti-cancer effects by modulating the PI3K/AKT signaling pathway, JadeAging may potentially achieve its anti-aging and antioxidant effects through the modulation of the insulin/IGF-1 and TOR pathways (Malek-Esfandiari et al., 2023). This study suggests that the expression of DAF-2 in *C. elegans* may significantly decrease on the ninth day after treatment with JadeAging. DAF-2, the insulin/insulin-like growth factor-1 (IGF-1) receptor in *C. elegans*, has been suggested to play a crucial role in regulating aging and lifespan. Downregulation of DAF-2 expression has been associated with a significant lifespan extension. Studies suggest that daf-2 long-lived mutants may live more than twice as long as normal worms, potentially because of reduced insulin/IGF-1 signaling, which activates the downstream DAF-16/FOXO transcription factor. DAF-16/FOXO initiates the expression of various genes linked to stress resistance, metabolism, and cellular protection, thereby contributing to the extension of lifespan (Melzer et al., 2020; Bansal et al., 2015). The TOR signaling pathway is a crucial regulator of cell growth, metabolism, and lifespan. It consists of two main complexes: mTORC1 the mammalian Target of Rapamycin Complex 1) and mTORC2 (mTOR Complex 2). mTORC1 is generally the primary focus of research on lifespan and health (Fontana et al., 2010). In *C. elegans*, RSKS-1 has been suggested to be the primary form of S6K. S6K is a direct downstream target of mTORC1 and its activation is thought to promote protein synthesis through the phosphorylation of ribosomal protein S6. Downregulation of RSKS-1 expression may imply reduced S6K activity, potentially decreasing protein synthesis and promoting a state conducive to increased lifespan (Fontana et al., 2010). mTORC1 negatively regulates autophagy by directly phosphorylating and inhibiting autophagy-related proteins such as ULK1 and ATG13. Downregulation of RSKS-1 expression may affect this mechanism, thereby altering the cellular response to nutrient deprivation and changes in the energy status (Laplante and Sabatini, 2009). In addition, studies suggest that combined mutations in daf-2 and rsks-1 may result in a nearly five-fold increase in lifespan, potentially surpassing the sum of the effects of individual mutations (Chen et al., 2013).

Autophagy is a well-conserved cellular degradation and recycling process that occurs in eukaryotic cells. Autophagy is thought to play a vital role in cell survival and homeostasis through the degradation of cytoplasmic organelles, proteins, and macromolecules (Parzych and Klionsky, 2014). Autophagy is a crucial process for maintaining cellular homeostasis and overall organismal health. Impaired autophagy is often associated with aging, potentially leading to the accumulation of damaged proteins and organelles, which may contribute to cellular dysfunction and development of age-related diseases (Laplante and Sabatini, 2009). Studies suggest that inhibition of TOR signaling may activate autophagy and potentially extend the lifespan of various model organisms (Aman et al., 2021). Our findings suggest that aging may promote autophagy through inhibition of the TOR signaling pathway, possibly *via* the suppression of RSKS-1 expression (Parzych and Klionsky, 2014). Furthermore, the worm homolog of cathepsin B, CPR-1, was significantly upregulated on the second day in JadeAging-treated animals. Based on the classification of proteins, CPR-1, as a cathepsin B-like protease, is commonly associated with intracellular waste processing, and such proteases typically participate in degradation processes related to endocytosis and autophagy (Xu et al., 2014). Enhanced expression of CPR-1 is thought to increase autophagic activity, potentially facilitating the clearance of damaged proteins and organelles within cells and maintaining intracellular homeostasis. This process may contribute to slowing the aging process and improving cell survival and function.

Antioxidant activity is thought to be one of the primary mechanisms by which JadeAging exerts its anti-aging effects. Oxidative stress is widely regarded as a critical factor driving cellular aging and increased frailty (Martemucci et al., 2022). This study suggests that JadeAging possesses antioxidant capacity, as evidenced by its protective effects against oxidative stress. JadeAging treatment appeared to significantly enhanced the ability of *C. elegans* to remain active after exposure to oxidants, suggesting its potential efficacy in combating oxidative stress. In addition, JadeAging may offer protection against age-related muscle decline (sarcopenia) to a degree comparable to that of the clinically validated urolithin A. RNA-seq data suggested that daf-2 expression may significantly decrease following JadeAging treatment. Studies suggest that in aged daf-2 mutants, preservation of muscle function is associated with improved muscle mitochondrial quality, maintained mitochondrial morphology, and higher intracellular ATP levels (Wang et al., 2019). These observations are consistent with the findings of the present study, indicating that JadeAging may exert its effects by reducing daf-2 expression. This study also evaluated the potential protective effects of Jade aging on mitochondrial morphology. Mitochondria maintain their function and quantity through a balance between fusion and fission. However, this balance may be disrupted during aging, leading to either excessively connected or fragmented mitochondrial networks that can impair normal mitochondrial function (Guo et al., 2023). These results suggest that on days 9 and 12 of adulthood, the JadeAging treatment group exhibited reduced mitochondrial fragmentation, which may indicate that JadeAging helps protect mitochondrial structure and potentially delays age-related mitochondrial morphological changes.

Taken together, our findings place JadeAging within a growing body of work using *C. elegans* as a whole-organism platform to evaluate natural antioxidants and plant-derived formulations. Like several polyphenol-rich extracts and traditional herbal preparations, JadeAging did not measurably extend lifespan at the doses tested, but it did improve multiple functional measures of health, including resistance to paraquat-induced oxidative stress, preservation of locomotor capacity, and maintenance of mitochondrial integrity and muscle structure. This pattern—robust healthspan benefits in the absence of overt lifespan extensions consistent with the idea that effective anti-aging interventions may primarily compress morbidity and preserve function rather than dramatically prolong survival.

From a translational perspective, these data suggest that JadeAging and its constituent extracts could be of interest as candidates for nutraceuticals, functional foods, or adjuvant health products aimed at supporting resilience to oxidative and mitochondrial stress, especially in the context of age-related decline in muscle function. The shared transcriptional signature involving pgp-1, gba-2, cyp-13A5, oac-14 and best-1 further points to coordinated regulation of xenobiotic transport, lysosomal and ion homeostasis, and metabolic detoxification. Although the present study was conducted in a nematode model, the convergence of functional and transcriptomic readouts provides a mechanistic rationale for further preclinical work in mammalian systems and, ultimately, for exploring whether JadeAging-based formulations can improve mobility, fatigue resistance, or other healthspan-related endpoints in humans.

Several limitations should be acknowledged when interpreting these findings. First, *C. elegans* is a powerful but inherently simplified model organism, and its physiology, pharmacokinetics, and exposure routes differ substantially from those of humans; therefore, direct extrapolation of dose and effect size is not appropriate. Second, our antioxidant assay used paraquat to induce oxidative stress and relied on locomotion as a functional readout, rather than directly quantifying intracellular reactive oxygen species or molecular markers of oxidative damage. Third, only a single genetic background, a limited dose range, and a defined set of phenotypic endpoints were examined, and we did not observe a consistent extension of lifespan, indicating that JadeAging should not be considered a “longevity drug” on the basis of the present data. Future work in additional nematode strains and mammalian models, combined with more detailed biochemical assays and, eventually, controlled clinical or functional food studies, will be necessary to determine whether the healthspan-related benefits observed here translate into meaningful improvements in human health and aging.

## 5 Conclusion

Animals were treated with JadeAging or one of its components (poria, ginseng, and rehmannia) and analyzed at both the early and later life stages using RNA-seq. These findings suggest that JadeAging may have a notable effect on the expression of genes related to nutrient sensing, stress-related pathways, and oxidation-reduction processes. The observed downregulation of daf-2 and rsks-1 and upregulation of cpr-1 following JadeAging treatment point to the possible activation of the TOR pathway and promotion of autophagy. The similarities in DEGs between the JadeAging and ginseng treatment groups suggested a potential overlap in their mechanisms of action. Further studies are required to fully elucidate the mechanisms of JadeAging and explore its potential therapeutic applications in age-related diseases. Although no significant positive effects were observed in the Vitality Platform, JadeAging and its components displayed antioxidant properties, protecting the animals from the harmful effects of paraquat. In addition, JadeAging appeared to provide protection against age-related muscle decline (sarcopenia), similar to the clinically validated urolithin A. Overall, these results support the potential health benefits of JadeAging and its components.

## Data Availability

The original contributions presented in the study are publicly available. This data can be found at the NCBI repository, accession number PRJNA1391804, available at: https://www.ncbi.nlm.nih.gov/sra/PRJNA1391804. Further inquiries can be directed to the corresponding author(s).
