# Supplementary material for: Antioxidant, antiaging and mitochondrial protective effects of JadeAging in Caenorhabditis elegans
Source: Front Pharmacol. 2026 Jan 12;16:1644921. doi: 10.3389/fphar.2025.1644921 (PMC12833404; doi:10.3389/fphar.2025.1644921)
Supplement: Supplementary file 1 [file Supplementaryfile1.pdf]

DEGs in common between JadeAging and Poria, Ginseng and Rehmannia at day 2.					
C. elegans gene	Description	Poria	Ginseng	Rehmannia	JadeAging
pgp-1	Multidrug resistance protein pgp-1	x	x	x	x
oac-14	O-ACyltransferase homolog	x	x	x	x
cyp-13A5	Putative cytochrome P450 CYP13A5	x	x	x	x
gba-2	Putative glucosylceramidase 2	x	x	x	x
clec-227	C-type LECtin		x	x	x
dod-21	Downstream Of DAF-16 (regulated by DAF-16)		x	x	x
T16G1.6	contains oxidoreductase and kinase-like domains		x	x	x
dhs-23	DeHydrogenases, Short chain			x	x
F55G11.8	Involved in innate immune response.			x	x
clec-57	C-type LECtin		x		x
cyp-14A4	CYtochrome P450 family		x		x
cyp-35A5	CYtochrome P450 family		x		x
dod-24	Downstream Of DAF-16 (regulated by DAF-16)		x		x
F13H6.3	Esterase CM06B1		x		x
C17H12.6	Involved in innate immune response.		x		x
C29F7.1	hypothetical protein		x		x
C40H1.8	Predicted to be involved in lipid metabolic process.		x		x
E02C12.10	hypothetical protein		x		x
F27D9.2	Predicted to enable transmembrane transporter activity.		x		x
F58B4.5	hypothetical protein		x		x
lbp-8	Lipid Binding Protein		x		x
ugt-16	UDP-GlucuronosylTransferase		x		x
ugt-21	UDP-GlucuronosylTransferase		x		x

DEGs in common between JadeAging and Poria, Ginseng and Rehmannia at day 9.					
C. elegans gene	Description	Poria	Ginseng	Rehmannia	JadeAging
best-24	BESTrophin (chloride channel) homolog	x	x	x	x
catp-3	Cation transporting ATPase		x	x	x
col-158	COLlagen		x	x	x
F18E3.12	hypothetical protein		x	x	x
mxl-3	MaX-Like		x	x	x
Y4C6B.2	Predicted to enable amino acid transmembrane transporter activity.		x	x	x
C49G7.7	hypothetical protein	x			x
ugt-51	UDP-GlucuronosylTransferase	x			x
asp-17	ASpartyl Protease			x	x
C31C9.7	hypothetical protein			x	x
clx-1	CoLlagen sequence X-hybridizing			x	x
F01D5.2	Contains ShK domain-like and ShKT domain			x	x
F41E6.5	Predicted to enable FMN binding activity and oxidoreductase activity.			x	x
ilys-2	Invertebrate LYSozyme			x	x
Y42G9A.3	Contains Leucine-rich repeat domain			x	x
acs-22	fatty Acid CoA Synthetase family		x		x
cpr-5	Cathepsin B-like cysteine proteinase 5		x		x
cyp-34A2	CYtochrome P450 family		x		x
fipr-22	FIP (Fungus-Induced Protein) Related		x		x
nit-1	NITrilase		x		x
pud-3	Protein Up-regulated in Daf-2(gf)		x		x
R03G8.6	Predicted to enable metalloaminopeptidase activity; peptide binding activity; and zinc ion binding activity.		x		x
rpr-1	ncRNA		x		x
Y4C6B.3	Predicted to enable transmembrane transporter activity.		x		x

JadeAging vs. Vehicle Control: Top 20 differentially downregulated genes at Day 2.			
C. elegans gene	Description	Fold-Change	*P*-Adj
gba-2	Putative glucosylceramidase 2	7.07446	<1E-10
T16G1.6	hypothetical protein	4.98067	<1E-10
ugt-21	UDP-GlucuronosylTransferase	3.98923	<1E-10
cyp-35A1	CYtochrome P450 family	3.97978	<1E-10
cyp-35A5	CYtochrome P450 family	3.85759	<1E-10
clec-206	C-type LECtin	3.69635	<1E-10
gst-5	Glutathione S-Transferase	3.03707	<1E-10
C40H1.8	hypothetical protein	2.96876	1.93E-08
B0024.4	hypothetical protein	2.83935	6.21E-08
oac-14	O-ACyltransferase homolog	2.72605	2.32E-08
lbp-8	Lipid Binding Protein	2.52342	2.90E-06
thn-1	THaumatiN family	2.51488	3.97E-06
cyp-35C1	CYtochrome P450 family	2.47404	8.24E-10
pgp-1	Multidrug resistance protein pgp-1	2.44937	1.28E-05
ugt-1	UDP-GlucuronosylTransferase	2.42049	6.34E-08
ugt-16	UDP-GlucuronosylTransferase	2.25572	7.12E-06
cyp-13A5	Putative cytochrome P450 CYP13A5	2.19876	1.96E-05
F20G2.5	hypothetical protein	2.18153	0.000369
C29F7.2	hypothetical protein	2.11717	1.60E-06
T25G12.13	hypothetical protein	2.06864	0.008649

JadeAging vs. Vehicle Control: Differentially downregulated genes at Day 2.			
C. elegans gene	Description	Fold-Change	*P*-Adj
dod-24	Downstream Of DAF-16 (regulated by DAF-16)	-2.05815	1.93E-03
dod-21	Downstream Of DAF-16 (regulated by DAF-16)	-2.0048	0.016888
C17H12.6	hypothetical protein	-1.99047	1.05E-02
F01D5.2	hypothetical protein	-1.97664	0.011441
hsp-16.2	Heat Shock Protein	-1.9618	2.66E-02
F55G11.8	hypothetical protein	-1.80486	0.007744

JadeAging vs. Vehicle Control: Differentially upregulated genes at Day 9.			
C. elegans gene	Description	Fold-Change	*P*-Adj
B0024.4	hypothetical protein	2.02857	2.68E-07
asp-17	ASpartyl Protease	1.95036	0.00000185
R02C2.7	hypothetical protein	1.77081	1.41E-03
ilys-2	Invertebrate LYSozyme	1.75606	0.000623
best-24	BESTrophin (chloride channel) homolog	1.63257	1.14E-04
rpr-1	ncRNA	1.5897	0.021335
C49G7.7	hypothetical protein	1.53916	5.88E-03
fbxa-145	F-box A protein	1.53863	0.003639
cpr-5	Cathepsin B-like cysteine proteinase 5	1.53698	2.61E-04
snpc-3.1	SNAPc (Small Nuclear RNA Activating Complex) homolog	1.51239	0.019568

JadeAging vs. Vehicle Control: Top 20 differentially downregulated genes at Day 9.			
C. elegans gene	Description	Fold-Change	*P*-Adj
comt-4	Catechol-O-MethylTransferase family	-2.24568	<1E-10
pud-3	Protein Up-regulated in Daf-2(gf)	-2.02646	1.28E-06
H37A05.4	hypothetical protein	-1.99537	1.81E-05
W06H8.6	hypothetical protein	-1.95247	1.81E-05
Y4C6B.3	hypothetical protein	-1.77629	6.90E-04
C09F9.2	hypothetical protein	-1.77002	2.61E-04
acs-22	fatty Acid CoA Synthetase family	-1.74352	8.03E-06
F18E3.12	hypothetical protein	-1.74238	1.41E-03
hpo-11	hypothetical protein	-1.74013	9.46E-05
gst-4	Glutathione S-transferase 4	-1.7166	2.53E-05
nit-1	NITrilase	-1.69961	3.64E-03
mxl-3	MaX-Like	-1.69123	1.96E-03
Y9D1A.1	hypothetical protein	-1.68188	4.39E-04
rle-1	Regulation of longevity by E3 ubiquitin-protein ligase	-1.6792	4.34E-05
cyp-34A2	CYtochrome P450 family	-1.67895	3.09E-03
K08D12.6	hypothetical protein	-1.66654	7.67E-03
Y51H4A.7	Probable urocanate hydratase	-1.65437	6.71E-03
unc-10	Rab-3-interacting molecule unc-10	-1.65405	0.009738
C32H11.4	hypothetical protein	-1.64816	9.74E-03
Y4C6B.2	hypothetical protein	-1.64406	0.003639
