# Supplementary material for: Antioxidant, antiaging and mitochondrial protective effects of JadeAging in Caenorhabditis elegans
Source: Front Pharmacol. 2026 Jan 12;16:1644921. doi: 10.3389/fphar.2025.1644921 (PMC12833404; doi:10.3389/fphar.2025.1644921)
Supplement: Supplementary file 2 [file Supplementaryfile2.docx]

**HPLC fingerprinting of the ginseng extract**

The fingerprint chromatogram of the ginseng extract was established by high-performance liquid chromatography (HPLC).

**Chromatographic conditions and system suitability**

An octadecylsilane-bonded silica column (InertSustain AQ-C18, 250 mm × 4.6 mm, 5 µm) was used as the stationary phase. The mobile phase consisted of solvent A (acetonitrile) and solvent B (aqueous solution), with gradient elution according to the program shown in Table Sx. The detection wavelength was 203 nm.

| Time (min) | Mobile phase A (%) | Mobile phase B (%) |
| --- | --- | --- |
| 0.00～10.00 | 1→3 | 99→97 |
| 10.00～20.00 | 3→7 | 97→93 |
| 20.00～30.00 | 7→8 | 93→92 |
| 30.00～40.00 | 8→14 | 92→86 |
| 40.00～62.00 | 14→20 | 86→80 |
| 62.00～70.00 | 20→25 | 80→75 |
| 70.00～80.00 | 25→30 | 75→70 |
| 80.00～90.00 | 30→33 | 70→67 |
| 90.00~91.00 | 33→35 | 67→65 |
| 91.00~110.00 | 35→45 | 65→55 |
| 110.00~120.00 | 45→95 | 55→5 |

**Preparation of reference and test solution**

Accurately 1.0 g of ginseng extract was weighed into a 100-mL conical flask, and approximately 25 mL of 50% (v/v) methanol–water was added. The mixture was sonicated for 60 min, allowed to cool to room temperature, the weight loss was made up with the same solvent, and the solution was filtered and mixed well. The filtrate was used as the sample solution.

**Assay procedure**

An aliquot of 10 µL of the sample solution was injected into the HPLC system, and the chromatogram was recorded to obtain the fingerprint.

**Reference chromatogram and major fingerprint peaks**

Retention times and peak areas of the major fingerprint peaks in the reference chromatogram were as follows:

**
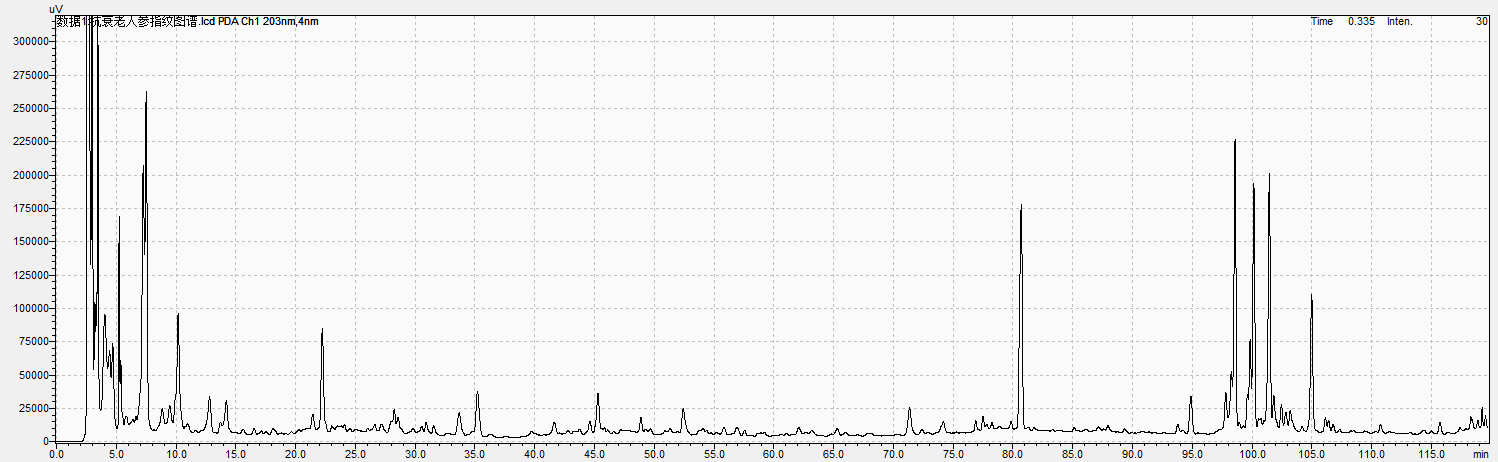
**

| Retention time (min) | Peak area |
| --- | --- |
| 22.231 | 1509598 |
| 80.696 | 2775053 |
| 98.583 | 2534813 |
| 100.150 | 2195841 |
| 101.44 | 2640767 |
| 104.986 | 151478 |

**Fingerprint similarity analysis**

The similarity index $S$between the chromatographic fingerprints was calculated according to the following equation:

$$S=\frac{\sum_{i-1}^{n} \left(Xbj-\mathop{X}\limits^{-} b\right)\left(Xsj-\mathop{X}\limits^{-} s\right)}{\sqrt{\sum_{i=1}^{n} \left(Xbj-\mathop{X}\limits^{-} b\right)^{2}}\sqrt{\sum_{i=1}^{n} \left(Xsj-\mathop{X}\limits^{-} s\right)^{2}}}$$

where $S$denotes the similarity of the two fingerprints, $n$is the number of common fingerprint peaks,$Xbj$ is the peak area of the $j$-th fingerprint peak in the chromatogram of the mixed extract，$\mathop{X}\limits^{-} b$ is the mean peak area of all fingerprint peaks in the mixed extract chromatogram，$Xsj$ is the peak area of the $j$-th fingerprint peak in the chromatogram of the standard extract，$\mathop{X}\limits^{-} s$ is the mean peak area of all fingerprint peaks in the standard extract chromatogram. The similarity index $S$was obtained from this calculation.

$$RDCki=\frac{\left|Xbki-Xski\right|}{Xski}$$

ve deviation of the characteristic component corresponding to the k-th fingerprint peak between the mixed extract and the standard extract. Xbki and Xski are the peak areas of the k-th fingerprint peak in the chromatograms of the mixed extract and the standard extract, respectively. RDCki is obtained according to this equation.

**Fingerprint chromatogram of the Rehmannia extract**

The fingerprint chromatogram of the Rehmannia extract was determined by high-performance liquid chromatography (HPLC).

**Chromatographic conditions and system suitability**

An octadecylsilane-bonded silica column (InertSustain AQ-C18, 250 mm × 4.6 mm, 5 μm) was used as the stationary phase. Acetonitrile was used as mobile phase A and an aqueous solution as mobile phase B. Gradient elution was performed according to the program shown in the table, and the detection wavelength was 203 nm.

| Time (min) | Mobile phase A (%) | Mobile phase A (%) |
| --- | --- | --- |
| 0.00～10.00 | 1→3 | 99→97 |
| 10.00～20.00 | 3→7 | 97→93 |
| 20.00～30.00 | 7→8 | 93→92 |
| 30.00～40.00 | 8→14 | 92→86 |
| 40.00～62.00 | 14→20 | 86→80 |
| 62.00～70.00 | 20→25 | 80→75 |
| 70.00～80.00 | 25→30 | 75→70 |
| 80.00～90.00 | 30→33 | 70→67 |
| 90.00~91.00 | 33→35 | 67→65 |
| 91.00~110.00 | 35→45 | 65→55 |
| 110.00~120.00 | 45→95 | 55→5 |

**Preparation of reference and test solutions**

Accurately 1.0 g of the Rehmannia extract was weighed into a 100-mL conical flask, and approximately 25 mL of 50% (v/v) methanol–water was added. The mixture was sonicated for 60 min, allowed to cool to room temperature, the loss in weight was made up with the same solvent, and the solution was filtered and mixed well. The filtrate was used as the sample solution.

**Assay**

An aliquot of 10 μL of the sample solution was injected into the HPLC system and the chromatogram was recorded to obtain the fingerprint.

**Reference chromatogram**

Retention times and peak areas of the major fingerprint peaks in the reference chromatogram were as follows:

**
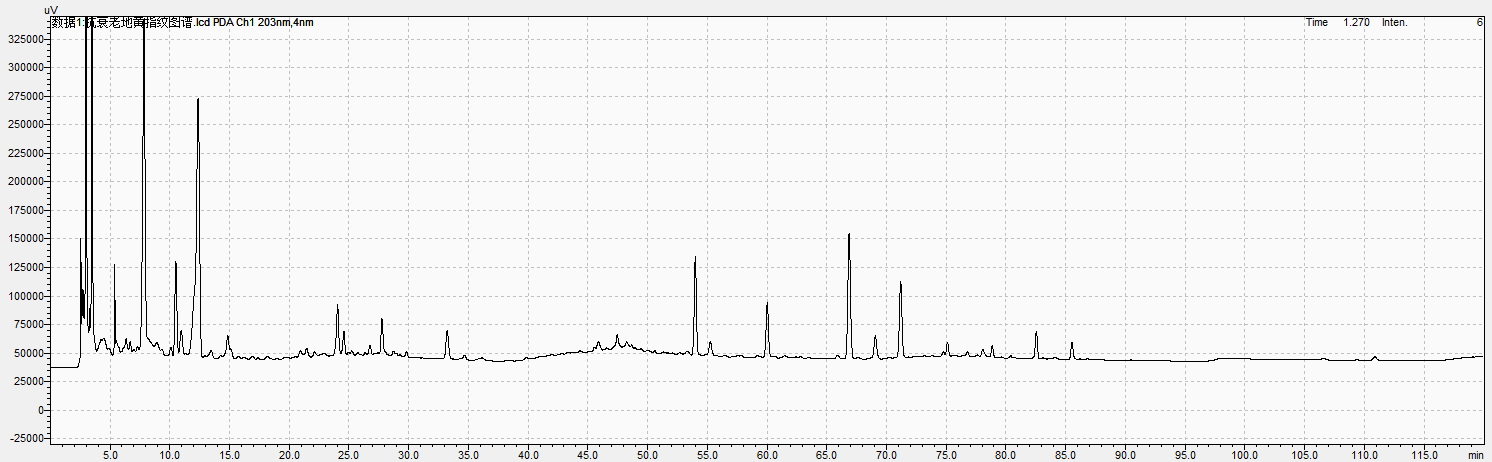
**

| Retention time (min) | Peak area |
| --- | --- |
| 12.377 | 5419962 |
| 24.046 | 885836 |
| 53.996 | 1288515 |
| 60.019 | 742627 |
| 66.877 | 1716331 |
| 71.190 | 1019730 |

**Fingerprint similarity analysis**

$$S=\frac{\sum_{i-1}^{n} \left(Xbj-\mathop{X}\limits^{-} b\right)\left(Xsj-\mathop{X}\limits^{-} s\right)}{\sqrt{\sum_{i=1}^{n} \left(Xbj-\mathop{X}\limits^{-} b\right)^{2}}\sqrt{\sum_{i=1}^{n} \left(Xsj-\mathop{X}\limits^{-} s\right)^{2}}}$$

where $S$denotes the similarity of the two fingerprints, $n$is the number of common fingerprint peaks,$Xbj$ is the peak area of the $j$-th fingerprint peak in the chromatogram of the mixed extract，$\mathop{X}\limits^{-} b$ is the mean peak area of all fingerprint peaks in the mixed extract chromatogram，$Xsj$ is the peak area of the $j$-th fingerprint peak in the chromatogram of the standard extract，$\mathop{X}\limits^{-} s$ is the mean peak area of all fingerprint peaks in the standard extract chromatogram. The similarity index $S$was obtained from this calculation.

$$RDCki=\frac{\left|Xbki-Xski\right|}{Xski}$$

ve deviation of the characteristic component corresponding to the k-th fingerprint peak between the mixed extract and the standard extract. Xbki and Xski are the peak areas of the k-th fingerprint peak in the chromatograms of the mixed extract and the standard extract, respectively. RDCki is obtained according to this equation.

**HPLC fingerprint of the Poria extract**

The fingerprint chromatogram of the Poria extract was determined by high-performance liquid chromatography (HPLC).

**Chromatographic conditions and system suitability**

An octadecylsilane-bonded silica column (InertSustain AQ-C18, 250 mm × 4.6 mm, 5 μm) was used as the stationary phase. Acetonitrile was used as mobile phase A and an aqueous solution as mobile phase B. Gradient elution was performed according to the program shown below, and the detection wavelength was 203 nm.

| Time (min) | Mobile phase A (%) | Mobile phase A (%) |
| --- | --- | --- |
| 0.00～10.00 | 1→3 | 99→97 |
| 10.00～20.00 | 3→7 | 97→93 |
| 20.00～30.00 | 7→8 | 93→92 |
| 30.00～40.00 | 8→14 | 92→86 |
| 40.00～62.00 | 14→20 | 86→80 |
| 62.00～70.00 | 20→25 | 80→75 |
| 70.00～80.00 | 25→30 | 75→70 |
| 80.00～90.00 | 30→33 | 70→67 |
| 90.00~91.00 | 33→35 | 67→65 |
| 91.00~110.00 | 35→45 | 65→55 |
| 110.00~120.00 | 45→95 | 55→5 |

**Preparation of reference and test solutions**

Accurately 1.0 g of the Poria extract was weighed into a 100-mL conical flask, and approximately 25 mL of 50% (v/v) methanol–water was added. The mixture was sonicated for 60 min, allowed to cool to room temperature, the loss in weight was made up with the same solvent, and the solution was filtered and mixed well. The filtrate was used as the sample solution.

**Assay**

An aliquot of 10 μL of the sample solution was injected into the HPLC system, and the chromatogram was recorded to obtain the fingerprint.

**Reference chromatogram**

Retention times and peak areas of the major fingerprint peaks in the reference chromatogram were as follows:

**
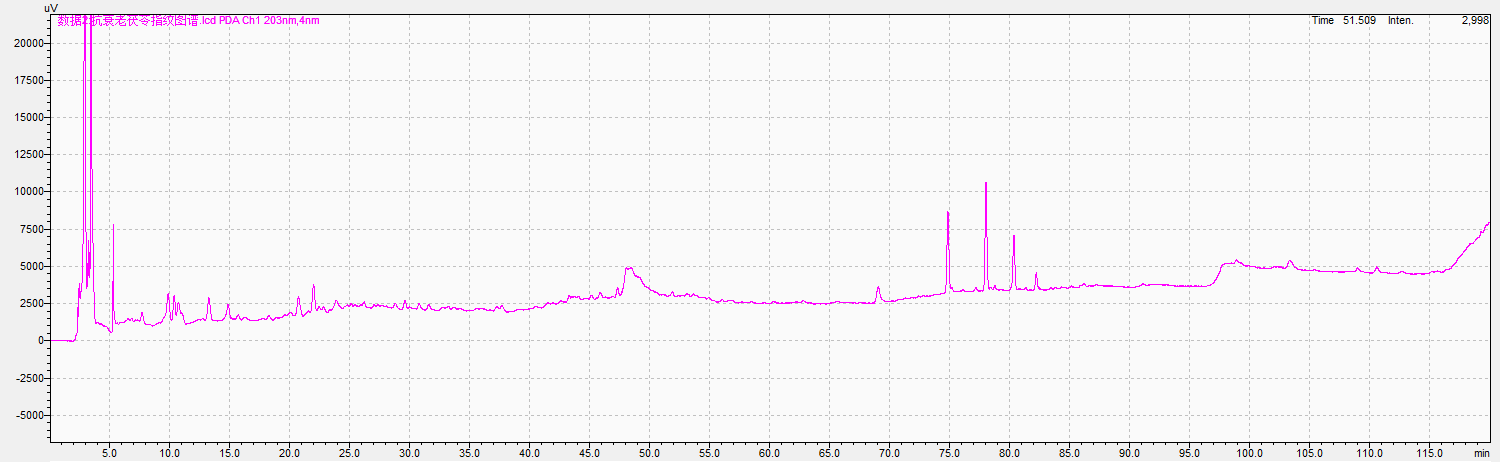
**

| Retention time (min) | Peak area |
| --- | --- |
| 74.869 | 65138 |
| 78.042 | 78464 |
| 80.002 | 40126 |

**Fingerprint similarity analysis**

$$S=\frac{\sum_{i-1}^{n} \left(Xbj-\mathop{X}\limits^{-} b\right)\left(Xsj-\mathop{X}\limits^{-} s\right)}{\sqrt{\sum_{i=1}^{n} \left(Xbj-\mathop{X}\limits^{-} b\right)^{2}}\sqrt{\sum_{i=1}^{n} \left(Xsj-\mathop{X}\limits^{-} s\right)^{2}}}$$

where $S$denotes the similarity of the two fingerprints, $n$is the number of common fingerprint peaks,$Xbj$ is the peak area of the $j$-th fingerprint peak in the chromatogram of the mixed extract，$\mathop{X}\limits^{-} b$ is the mean peak area of all fingerprint peaks in the mixed extract chromatogram，$Xsj$ is the peak area of the $j$-th fingerprint peak in the chromatogram of the standard extract，$\mathop{X}\limits^{-} s$ is the mean peak area of all fingerprint peaks in the standard extract chromatogram. The similarity index $S$was obtained from this calculation.

$$RDCki=\frac{\left|Xbki-Xski\right|}{Xski}$$

ve deviation of the characteristic component corresponding to the k-th fingerprint peak between the mixed extract and the standard extract. Xbki and Xski are the peak areas of the k-th fingerprint peak in the chromatograms of the mixed extract and the standard extract, respectively. RDCki is obtained according to this equation.

**HPLC fingerprint of JadeAging**

The fingerprint chromatogram of JadeAging was determined by high-performance liquid chromatography (HPLC).

**Chromatographic conditions and system suitability**

An octadecylsilane-bonded silica column (InertSustain AQ-C18, 250 mm × 4.6 mm, 5 μm) was used as the stationary phase. Acetonitrile was used as mobile phase A and an aqueous solution as mobile phase B. Gradient elution was performed according to the program shown below, and the detection wavelength was 203 nm.

| Time (min) | Mobile phase A (%) | Mobile phase A (%) |
| --- | --- | --- |
| 0.00～10.00 | 1→3 | 99→97 |
| 10.00～20.00 | 3→7 | 97→93 |
| 20.00～30.00 | 7→8 | 93→92 |
| 30.00～40.00 | 8→14 | 92→86 |
| 40.00～62.00 | 14→20 | 86→80 |
| 62.00～70.00 | 20→25 | 80→75 |
| 70.00～80.00 | 25→30 | 75→70 |
| 80.00～90.00 | 30→33 | 70→67 |
| 90.00~91.00 | 33→35 | 67→65 |
| 91.00~110.00 | 35→45 | 65→55 |
| 110.00~120.00 | 45→95 | 55→5 |

**Preparation of reference and test solutions**

Accurately 1.0 g of JadeAging was weighed into a 100-mL conical flask, and approximately 25 mL of 50% (v/v) methanol–water was added. The mixture was sonicated for 60 min, allowed to cool to room temperature, the loss in weight was made up with the same solvent, and the solution was filtered and mixed well. The filtrate was used as the sample solution.

**Assay**

An aliquot of 10 μL of the sample solution was injected into the HPLC system, and the chromatogram was recorded to obtain the fingerprint.

**Reference chromatogram**

Retention times and peak areas of the major fingerprint peaks in the reference chromatogram were as follows:

**
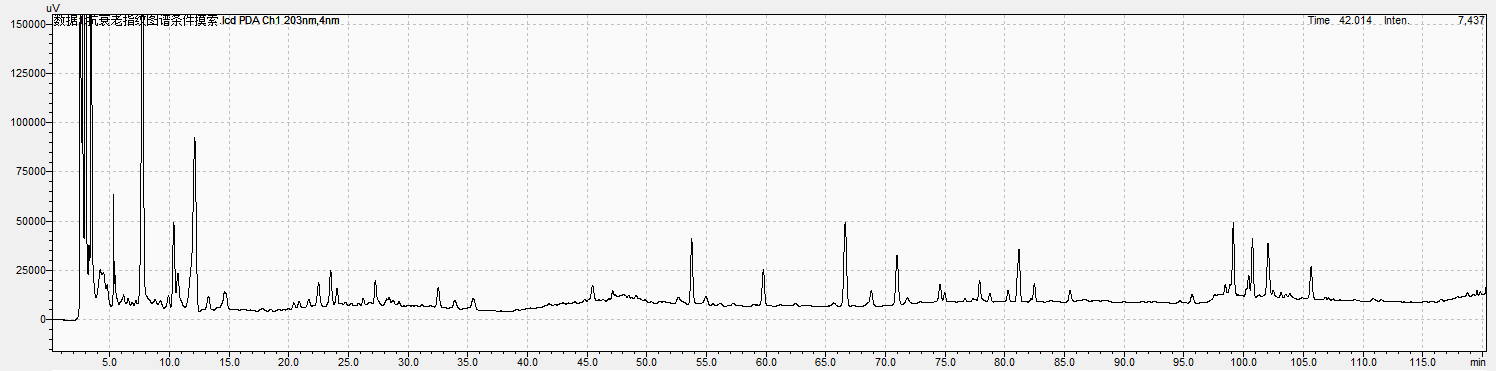
**

| Retention time (min) | Peak area |
| --- | --- |
| 12.122 | 2186225 |
| 23.513 | 466448 |
| 53.762 | 437116 |
| 59.762 | 279879 |
| 66.620 | 675192 |
| 70.963 | 388503 |
| 77.879 | 168580 |
| 80.696 | 2775053 |
| 98.583 | 2534813 |
| 100.153 | 2195841 |
| 101.444 | 2640767 |
| 104.986 | 1514178 |

**Fingerprint similarity analysis**

$$S=\frac{\sum_{i-1}^{n} \left(Xbj-\mathop{X}\limits^{-} b\right)\left(Xsj-\mathop{X}\limits^{-} s\right)}{\sqrt{\sum_{i=1}^{n} \left(Xbj-\mathop{X}\limits^{-} b\right)^{2}}\sqrt{\sum_{i=1}^{n} \left(Xsj-\mathop{X}\limits^{-} s\right)^{2}}}$$

where $S$denotes the similarity of the two fingerprints, $n$is the number of common fingerprint peaks,$Xbj$ is the peak area of the $j$-th fingerprint peak in the chromatogram of the mixed extract，$\mathop{X}\limits^{-} b$ is the mean peak area of all fingerprint peaks in the mixed extract chromatogram，$Xsj$ is the peak area of the $j$-th fingerprint peak in the chromatogram of the standard extract，$\mathop{X}\limits^{-} s$ is the mean peak area of all fingerprint peaks in the standard extract chromatogram. The similarity index $S$was obtained from this calculation.

$$RDCki=\frac{\left|Xbki-Xski\right|}{Xski}$$

ve deviation of the characteristic component corresponding to the k-th fingerprint peak between the mixed extract and the standard extract. Xbki and Xski are the peak areas of the k-th fingerprint peak in the chromatograms of the mixed extract and the standard extract, respectively. RDCki is obtained according to this equation.
